# Maternal diet alters long-term innate immune cell memory in fetal and juvenile hematopoietic stem and progenitor cells in nonhuman primate offspring

**DOI:** 10.1016/j.celrep.2023.112393

**Published:** 2023-04-13

**Authors:** Michael J. Nash, Evgenia Dobrinskikh, Taylor K. Soderborg, Rachel C. Janssen, Diana L. Takahashi, Tyler A. Dean, Oleg Varlamov, Jon D. Hennebold, Maureen Gannon, Kjersti M. Aagaard, Carrie E. McCurdy, Paul Kievit, Bryan C. Bergman, Kenneth L. Jones, Eric M. Pietras, Stephanie R. Wesolowski, Jacob E. Friedman

**Affiliations:** 1 Department of Pediatrics, University of Colorado Anschutz Medical Campus, Aurora, CO 80045, USA; 2 Harold Hamm Diabetes Center, University of Oklahoma Health Sciences Center, Oklahoma City, OK 73104, USA; 3 Division of Cardiometabolic Health, Oregon National Primate Research Center, Oregon Health & Science University, Beaverton, OR 97006, USA; 4 Division of Reproductive and Developmental Sciences, Oregon National Primate Research Center, Oregon Health & Science University, Beaverton, OR 97006, USA; 5 Department of Medicine, Division of Diabetes, Endocrinology, and Metabolism, Vanderbilt University Medical Center, Nashville, TN 37235, USA; 6 Department of Obstetrics and Gynecology, Division of Maternal-Fetal Medicine, Baylor College of Medicine, Houston, TX 77030, USA; 7 Department of Human Physiology, University of Oregon, Eugene, OR 97403, USA; 8 Department of Medicine, University of Colorado Anschutz Medical Campus, Aurora, CO 80045, USA; 9 Department of Physiology, University of Oklahoma Health Sciences Center, Oklahoma City, OK 73104, USA; 10 Senior author; 11 Lead contact

## Abstract

Maternal overnutrition increases inflammatory and metabolic disease risk in postnatal offspring. This constitutes a major public health concern due to increasing prevalence of these diseases, yet mechanisms remain unclear. Here, using nonhuman primate models, we show that maternal Western-style diet (mWSD) exposure is associated with persistent pro-inflammatory phenotypes at the transcriptional, metabolic, and functional levels in bone marrow-derived macrophages (BMDMs) from 3-year-old juvenile offspring and in hematopoietic stem and progenitor cells (HSPCs) from fetal and juvenile bone marrow and fetal liver. mWSD exposure is also associated with increased oleic acid in fetal and juvenile bone marrow and fetal liver. Assay for transposase-accessible chromatin with sequencing (ATAC-seq) profiling of HSPCs and BMDMs from mWSD-exposed juveniles supports a model in which HSPCs transmit pro-inflammatory memory to myeloid cells beginning *in utero*. These findings show that maternal diet alters long-term immune cell developmental programming in HSPCs with proposed consequences for chronic diseases featuring altered immune/inflammatory activation across the lifespan.

## In brief

Using nonhuman primate models, Nash et al. show that maternal Western-style diet provokes reprogramming of fetal hematopoietic stem and progenitor cells to transmit pro-inflammatory memory to myeloid cells for years after weaning, even when offspring are fed a conventional diet, predisposing these offspring to inflammatory disease across the lifespan.

## Graphical abstract

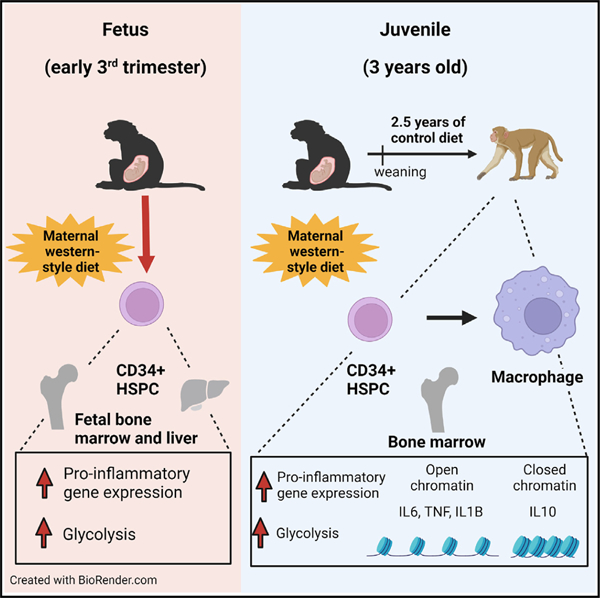

## INTRODUCTION

Maternal stressors and other environmental factors affect the developing embryo and fetus in ways that lead to increased susceptibility for chronic disease in later life. Maternal obesity and Western-style diet (WSD) exposure are linked with increased risk for a number of childhood and adult inflammation-related disorders, including obesity, diabetes, thyroid disorders, cardiovascular disease, and nonalcoholic fatty liver disease (NAFLD), independent of genetic and lifestyle determinants.^1–10^ Developmental programming of chronic low-grade inflammation plays an important role in the onset and progression of these diseases.^11,12^

Emerging evidence suggests a link between maternal obesogenic environment and fetal immune cell reprogramming.^13,14^ Innate immune cells, including macrophages and monocytes, can form an immune “memory” of previous stimuli, which is associated with higher baseline pro-inflammatory activity and/or response to secondary insults for the lifespan of the cell.^15,16^ The establishment of innate immune cell memory involves increased glycolysis, reduced oxidative phosphorylation, and expression of transcription factors, including FOS/JUN and CCAAT/enhancer binding protein β (C/EBPβ), which primes for pro-inflammatory activity.^16,17^ Furthermore, this memory relies on the propagation of epigenetic modifications that develop in hematopoietic stem and progenitor cells (HSPCs), which can be passed on to progeny immune cells (i.e., macrophages).^16–19^ Fetal hematopoietic function is sensitive to maternal WSD (mWSD) exposure and mWSD decreases HSPC renewal while potentiating HSPC-based myeloid differentiation in the murine fetal liver.^20^ In addition, fetal peripheral blood mononuclear cells (MNCs) in a baboon model of maternal obesity showed transcriptional changes in immune cell function pathways.^21^ Further, in mice and a macaque model of mWSD, macrophages and splenocytes from offspring weaned to a control diet exhibited increased tumor necrosis factor alpha (TNFα) responses to lipopolysaccharide (LPS).^22–24^ In mice, prenatal exposure to inflammation triggered a transient increase in lymphoid-biased fetal HSPCs and functional changes in postnatal hematopoiesis and immune output.^25^ Likewise, in adult mice, obesity induced persistent epigenomic reprogramming of macrophages toward enhanced angiogenic and inflammatory responses.^26^ These studies suggest that immune cells are programmed by *in utero* exposure to mWSD via an as-yet characterized mechanism. It is also unclear whether HSPC or immune cell responses in association with mWSD exposure *in utero* represent transient effects, or whether they represent the early origins of developmentally programmed inflammation that persists later in life.

Here, we leveraged a well-characterized model of mWSD exposure in macaques,^24,27–34^ which recapitulates complex features of immune cell development present in humans,^35^ to test whether mWSD is associated with a pro-inflammatory macrophage phenotype in juvenile offspring at 3 years of age. We hypothesized that mWSD exposure would produce a pro-inflammatory phenotype in fetal HSPCs, which would persist in juvenile HSPCs, leading to bone marrow-derived macrophage (BMDM) progeny cells that share this pro-inflammatory phenotype postnatally. Our data demonstrate that mWSD provokes reprogramming of the transcriptional and metabolic profile of offspring HSPCs *in utero* and myeloid cells show less oxidative phosphorylation and a shift to glycolysis, ultimately leading to pro-inflammatory cytokine transcription. These changes persist with altered epigenetic regulation for years after weaning, even when offspring are fed a conventional diet, thus predisposing these offspring to inflammatory disease across the lifespan.

## RESULTS

### Impact of WSD on maternal, fetal, and juvenile macaque metabolic phenotypes

Fetuses were studied in the early third trimester from rhesus macaque dams fed a chronic WSD or standard monkey chow control diet (CD) for 5.5 years prior to pregnancy as described previously.^36,37^ WSD-fed dams had higher percentage body fat than CD-fed dams but had normal insulin and glucose levels (Table S1). The areas under the curve for insulin and glucose from an intravenous glucose tolerance test (IVGTT) during pregnancy were not different in WSD-fed dams vs. CD-fed dams (Table S1). Fetuses exposed to mWSD had no differences in body weight, crown-rump length, liver weight, retroperitoneal white adipose tissue weight, or blood glucose concentrations compared with maternal CD (mCD)-exposed fetuses (Table S1).

Three-year-old juvenile offspring were studied from Japanese macaque dams fed a chronic WSD or CD as described previously.^32^ WSD-fed dams were obese prior to pregnancy but had normal glucose and insulin levels during pregnancy (Table S2). All juveniles were weaned to the CD at 7 months of age and fed the CD for about 2.5 years. Juveniles exposed to mWSD during gestation and lactation had increased body weight, lean mass, and retroperitoneal white adipose tissue mass but no change in percentage body fat (Table S2). No differences were observed in fasting glucose and insulin or the area under the curve for insulin and glucose during IVGTT between the two juvenile groups. No differences were observed between male and female offspring for these phenotypic data (p > 0.05) and thus, in all subsequent data, results are shown with a mix of both male and female offspring.

### mWSD-exposed juvenile BMDMs have a pro-inflammatory phenotype

**Figure 1.**
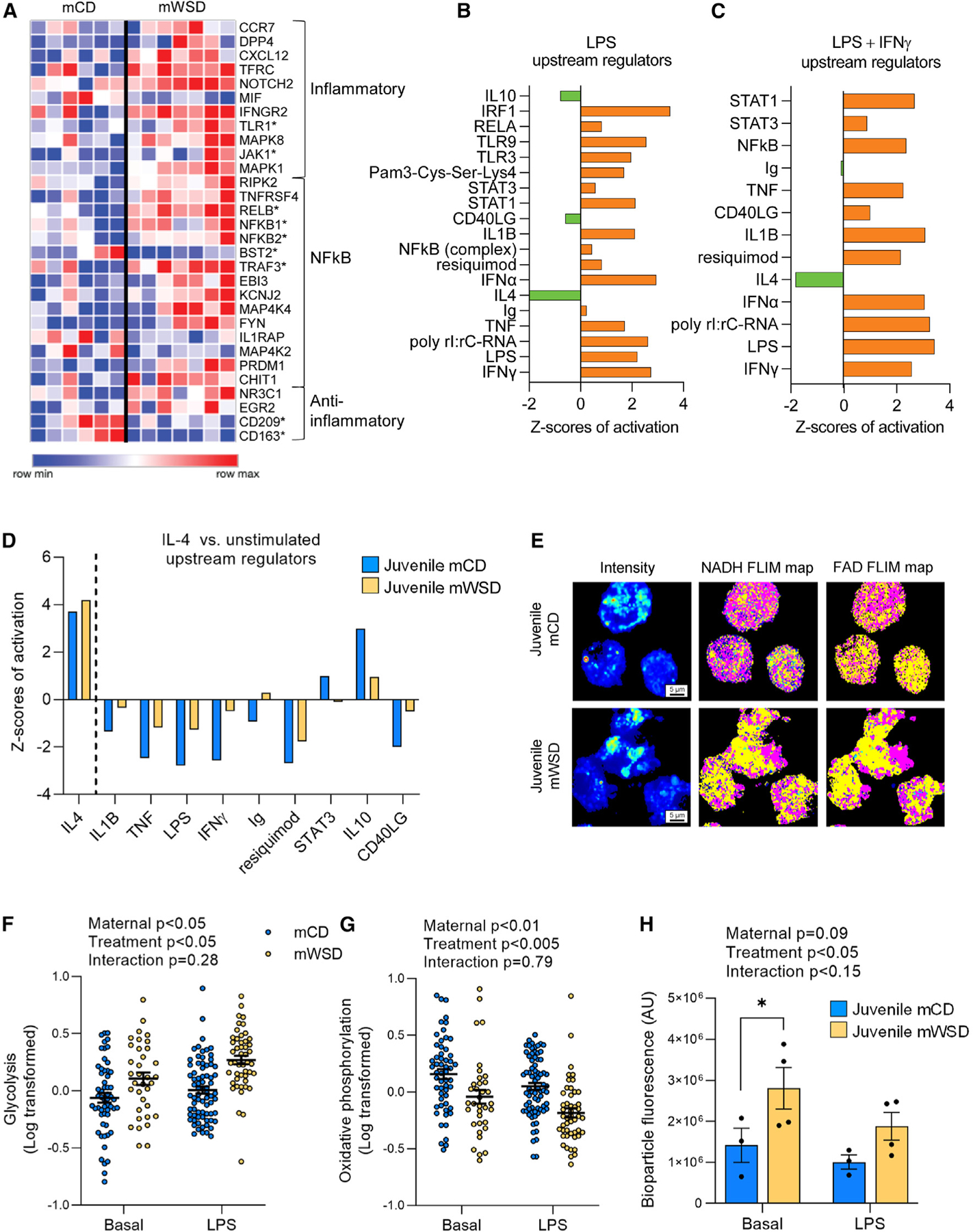
Juveniles exposed to mWSD have BMDMs with increased functional pro-inflammatory gene expression profiles (A) Heatmap of DEGs from NanoString analysis classified as inflammatory, NF-κB-related, and anti-inflammatory pathways in unstimulated BMDMs. All genes shown are significantly different vs. mCD. Heatmap with genes grouped by functional category. One offspring per column. (B and C) Activation *Z* scores for upstream regulators derived from comparison of mCD- vs. mWSD-exposed BMDM gene expression response to LPS (B) and LPS + IFNγ (C). Orange bars indicate positive *Z* score and upregulation; green bars, negative *Z* score and downregulation. Pathways shown are significantly different between mCD vs. mWSD. (D) Activation *Z*-scores for predicted upstream regulators comparing unstimulated gene expression vs. IL-4-stimulated gene expression in mCD-exposed (blue) and mWSD-exposed (yellow) BMDMs. Upstream regulators shown are different (p < 0.05) in both mCD- and mWSD-exposed BMDMs. (E–G) FLIM maps of juvenile BMDMs (E). For FLIM intensity maps, red and yellow indicate higher intensity of fluorescence lifetimes and blue and green indicate lower intensity of fluorescence lifetimes. For NADH maps, yellow indicates free NADH and pink indicates bound NADH. For FAD maps, yellow indicates bound FAD and pink indicates free FAD. Glycolysis (F) and oxidative phosphorylation (G) in mCD- and mWSD-exposed BMDMs at baseline and in response to LPS stimulation. (F and G) Each dot represents one cell; 20–30 cells used per animal. (H) Phagocytosis of *E. coli* biospheres in BMDMs at baseline and in response to LPS. Data are mean ± SEM (F–H). p values for effect of mWSD, effect of LPS treatment, and interaction between mWSD and treatment were calculated by repeated measures two-way ANOVA with log-transformed data (F–H) and *p < 0.05, Fisher’s least significant difference (LSD) post-test comparison (H). n = 4–6 mCD, n = 3–7 mWSD.

To determine long-term effects of mWSD exposure on the macrophage inflammatory phenotype in offspring, we isolated bone marrow cells from juveniles, differentiated the MNCs into BMDMs, and measured gene expression in BMDMs from mCD- vs. mWSD-exposed juveniles at baseline and following stimulation with LPS (Toll-like receptor [TLR]-4 agonist), LPS + interferon gamma (IFNγ) (polarizes macrophages to a pro-inflammatory phenotype),^38^ or interleukin (IL)-4 (polarizes macrophages to a reparative phenotype).^38,39^ At baseline (unstimulated), 66 differentially expressed genes (DEGs) in juvenile BMDMs from mWSD- vs. mCD-exposed offspring were observed, with 36 upregulated and 30 downregulated (Figure S1). Notably, we observed a baseline upregulation in the expression of genes associated with pro-inflammatory macrophage signaling pathways, including *TLR1* and *JAK1*, and genes associated with the nuclear factor κB (NF-κB) pathway, including *NFKB1*, *NFKB2*, and *RELB* (Figure 1A). Conversely, the expression of genes associated with an anti-inflammatory macrophage phenotype (*CD209*, *CD163*) was downregulated, whereas the demethylase EGR2, which links M2 polarization signals to the epigenome,^40^ was upregulated (Figure 1A).

Next, in response to stimulation with pro-inflammatory TLR agonists, we identified putative upstream regulators (as defined by ingenuity pathway analysis [IPA]) derived from DEGs in BMDMs from mWSD- vs. mCD-exposed juveniles. In response to LPS, upstream regulators predicted to be upregulated included IFNγ, LPS, NF-κB complex, STAT1, STAT3, TLR3, and TLR9 in mWSD- vs. mCD-exposed BMDMs, whereas upstream regulators associated with resolution of inflammation, including IL10, CD40LG, and IL4, were predicted to be downregulated (Figure 1B). In response to LPS + IFNγ, BMDMs from mWSD-exposed juveniles had further upregulation of pro-inflammatory regulators such as STAT1, STAT3, NF-κB, IFNγ, TNF, and IL1B compared with mCD-exposed BMDMs (Figure 1C).

To examine whether mWSD exposure compromised the anti-inflammatory response, BMDMs were stimulated with IL-4. The profile (identity) of predicted upstream regulators (as defined by IPA) with IL-4 stimulation was similar between mWSD and mCD groups. As expected, IL4 was the top predicted upstream regulator in both groups; however, compared with mCD, BMDMs from mWSD-exposed juveniles had less robust IL-4-mediated downregulation of the pro-inflammatory regulators IL1B, TNF, LPS, and IFNγ (Figure 1D). Overall, BMDMs from mWSD-exposed juveniles demonstrated higher baseline pro-inflammatory gene expression, increased inflammatory response to TLR agonists, and blunted gene expression responses to anti-inflammatory IL-4 stimulation.

One signature of pro-inflammatory macrophages is a metabolic shift toward increased glycolysis and a concurrent reduction in oxidative phosphorylation.^41^ We used fluorescence lifetime imaging microscopy (FLIM) to measure glycolysis (glycolytic index) and oxidative phosphorylation (fluorescence lifetime redox ratio [FLIRR]).^42–44^ The intrinsic autofluorescence lifetimes of free and enzyme-bound NADH and flavin adenine dinucleotide (FAD) were measured as illustrated in Figure 1E. mWSD-exposed live BMDMs had increased glycolysis (maternal p < 0.05; Figure 1F) and reduced oxidative phosphorylation (maternal p < 0.01; Figure 1G) at baseline. In response to LPS, glycolysis was increased (treatment p < 0.05; Figure 1F) and oxidative phosphorylation was decreased (treatment p < 0.005; Figure 1G) across diet groups, consistent with a metabolic shift away from oxidative metabolism to glycolysis.

Macrophages engulf bacteria in association with elevated pro-inflammatory molecules and glycolysis.^45^ Here, we assessed the ability of BMDMs from mWSD- and mCD-exposed juveniles to phagocytose *Escherichia coli* bioparticles. At baseline, phagocytosis of *E. coli* bioparticles was increased by 45% in BMDMs from mWSD-exposed juveniles compared with mCD-exposed juveniles (p < 0.05, post-test; Figure 1H). In response to LPS, phagocytosis exhibited a similar trend, although levels were not significantly different between the groups (p = 0.16, post-test; Figure 1H), suggesting that the major effects of mWSD were on basal levels of phagocytosis, consistent with the measured gene expression patterns. Overall, mWSD caused a persistent increase in pro-inflammatory transcriptional, metabolic, and functional activities in BMDMs that persisted into juvenile life.

### HSPCs have a pro-inflammatory phenotype in juveniles exposed to mWSD during gestation and lactation

**Figure 2.**
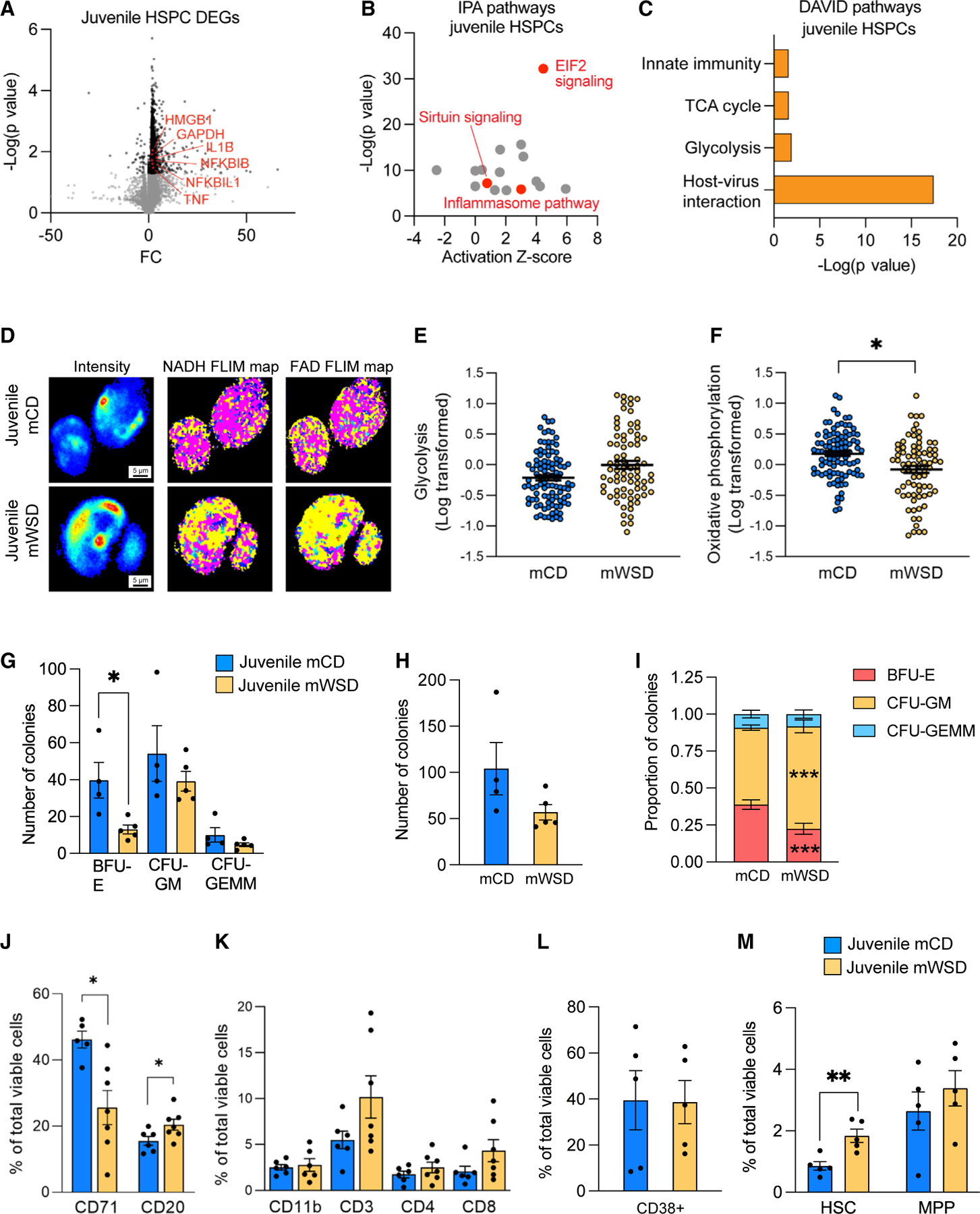
Bone marrow HSPCs from juveniles exposed to mWSD have a pro-inflammatory metabolic and transcriptional phenotype (A) Volcano plot of –Log(p value) and fold change (FC) of DEGs in mWSD- vs. mCD-exposed juvenile HSPCs. Key upregulated genes are highlighted in red. Gray circles indicate genes with p > 0.05. Black circles indicate genes with p < 0.05 in mWSD vs. mCD, regardless of whether they passed quality check measures and thus regardless of whether they featured in the 1,508 DEGs used for subsequent analyses. All genes highlighted in red passed quality check measures and were included in the 1,508 DEGs. n = 6 mCD, n = 6 mWSD. (B) Plot of IPA-derived canonical pathways from juvenile HSPC DEGs. Red dots indicate pathways of interest and gray dots indicate other pathways described in Table S3. (C) Highlighted pathways from DAVID analysis from juvenile HSPC DEGs. (D–F) FLIM map of juvenile bone marrow HSPCs (D). For FLIM intensity maps, red and yellow indicate higher intensity of fluorescence lifetimes and blue and green indicate lower intensity of fluorescence lifetimes. For NADH maps, yellow indicates free NADH and pink indicates bound NADH. For FAD maps, yellow indicates bound FAD and pink indicates free FAD. Glycolysis (E) and oxidative phosphorylation (F) in mCD- and mWSD-exposed bone marrow HSPCs. (E and F) Each dot represents one HSPC; 7–20 cells used per animal. (G–I) CFU assays of juvenile bone marrow MNCs. Colony count from mCD- and mWSD-exposed bone marrow MNCs (G). Total number of colonies grown from mCD- and mWSD-exposed bone marrow MNCs (H). Proportion of CFU-GEMM, CFU-GM, and BFU-E colonies (I). Proportion of colonies was calculated by dividing the total count for each colony type per animal by the total colony number per animal. These calculations were input as n = 1. n = 4 mCD, n = 5 mWSD. (J–M) Flow cytometry analysis of juvenile bone marrow MNCs. Juvenile CD71+ cells and CD20+ B cells as a percentage of total viable cells (J). Overall bone marrow CD11b+ myeloid cells and CD3+, CD4+, and CD8+ T cells as a percentage of total viable cells (K). CD38+ cells (L) and HSCs (CD34+CD38−CD45RA−CD90+) and MPPs (CD34+CD38−CD45RA−CD90−) (M) as a percentage of total viable CD34+ cells in mCD (blue) and mWSD (yellow) juveniles. n = 5–6 mCD, n = 5–7 mWSD. Data are mean ± SEM (E–M). *p < 0.05, **p < 0.01, ***p < 0.001, mWSD effect from repeated measures ANOVA with log-transformed data (E–F), unpaired Student’s t test (G–I), or Mann-Whitney U test (J–M).

HSPCs are the long-term reservoir for short-lived immune cells, including macrophages. Therefore, we assessed whether HSPCs exhibit similar pro-inflammatory features to BMDMs that may be inherited by BMDMs. We performed bulk RNA sequencing (RNA-seq) in column-enriched bone marrow CD34+ cells (hereafter defined as HSPCs) from mWSD- and mCD-exposed juveniles. Principal-component analysis (PCA) separated mWSD- and mCD-exposed HSPCs (Figure S2). In total, we identified 1,508 DEGs between the two conditions, with the majority being upregulated (Figure 2A). Similar to mWSD-exposed BMDMs, we observed gene expression patterns consistent with a pro-inflammatory phenotype in mWSD-exposed HSPCs, including *IL1B*, *TNF*, *NFKBIB*, and *NFKBIL1* (Figure 2A). Likewise, we observed upregulation in genes associated with glycolytic metabolism (*GAPDH*, *HMGB1*) (Figure 2A). We next determined the putative canonical pathways and upstream regulators enriched in these DEGs in juvenile HSPCs. Key upregulated pathways in HSPCs from mWSD-exposed juveniles included the inflammasome pathway (which relates to IL-1β signaling), sirtuin signaling, and EIF2, a major translational regulator (Figure 2B). The complete lists of canonical pathways and upstream regulators derived from IPA are presented in Tables S3 and S4, respectively. To corroborate these findings, we performed Gene Ontology (GO) analyses of the DEGs using DAVID. We identified a similar pattern of pathway enrichment (Figure 2C), including gene signatures for innate immunity, tricarboxylic acid (TCA) cycle, glycolysis, and host-virus interactions, which is a pathway composed of inflammation-associated genes. Other pathways related to DNA damage, protein synthesis, and ribosome assembly were also identified in both IPA and DAVID analyses (see Tables S3 and S4 for IPA analyses). To determine whether these transcriptional signatures were associated with functional differences in mWSD-exposed HSPCs, we used FLIM (Figure 2D) in HSPCs from mWSD-exposed juveniles, which showed a trending increase in glycolysis (p = 0.11) (Figure 2E) and a significant 25% reduction (p < 0.05) in oxidative phosphorylation compared with mCD-exposed HSPCs (Figure 2F). These metabolic effects on glycolysis and oxidative phosphorylation were consistent with the gene expression signatures and pro-inflammatory pathway activation in mWSD-exposed HSPCs and BMDMs from juveniles.

We next assessed whether mWSD exposure might exert latent effects on the differentiation potential of the bone marrow using colony-forming unit (CFU) assays with isolated bone marrow MNCs. Strikingly, in juveniles exposed to mWSD 2.5 years earlier, the number of burst forming unit-erythroid (BFU-E) colonies was decreased over 2-fold, whereas colony counts of CFU-granulocyte/monocyte (CFU-GM) and CFU-granulocyte/monocyte/megakaryocyte/erythrocyte (CFU-GEMM) were unchanged (Figure 2G). In bone marrow MNCs from juveniles, no difference in the total number of colonies formed in mWSD-exposed vs. mCD-exposed cells was observed (Figure 2H); however, the proportion of CFU-GM was increased (Figure 2I). Despite these differences in bone marrow clonogenicity, no differences were seen in complete blood cell counts (CBCs) between mWSD- and mCD-exposed juveniles (Table S4). In the murine fetal liver, maternal obesity together with a maternal high-fat diet skewed HSPCs toward myeloid-biased differentiation that was niche dependent.^20^ This suggests that chronic high-fat diet biases the microenvironment (associated with oxidative stress), favoring HSPCs toward myeloid differentiation at the expense of hematopoietic stem cell (HSC) self-renewal. Overall, these data support a shift in the output of bone marrow differentiation capacity toward increased myeloid production at the expense of the erythroid lineage output due to mWSD.

Given the shift in clonogenic potential, we used flow cytometry to determine whether mWSD exposure increased myeloid cell frequency in the juvenile bone marrow, as reported with postnatal WSD consumption.^46^ In line with our CFU assays, bone marrow MNCs from mWSD-exposed juveniles exhibited a significant reduction in the frequency of CD71+ erythroid progenitor cells compared with mCD-exposed offspring, whereas the frequency of CD20+ B cells was modestly but significantly increased (Figure 2J). On the other hand, no change in the frequencies of CD11b+ myeloid cells and CD3+, CD4+, and CD8+ T cells was observed (Figure 2K). We next characterized the immature bone marrow HSPC compartment in juveniles using flow cytometry. Gating strategies and representative flow cytometry plots are shown in Figure S3. With no change in CD34+CD38+ cells (Figure 2L), we noted a 2-fold increase in phenotypic HSCs (CD34+CD38−CD45RA−CD90+) within the CD34+ HSPC compartment of mWSD-exposed juveniles compared with mCD-exposed offspring but no change in the frequency of multipotent progenitor cells (MPPs; CD34+CD38−CD45RA−CD90−) (Figure 2M). Taken together, these results demonstrate that, similar to BMDMs, HSPCs from juveniles exposed to mWSD, even when weaned to CD for 2.5 years, have transcriptional, functional, and metabolic signatures consistent with pro-inflammatory activation.

### Increased chromatin accessibility at pro-inflammatory genes in HSPCs and BMDMs from juveniles exposed to mWSD

**Figure 3.**
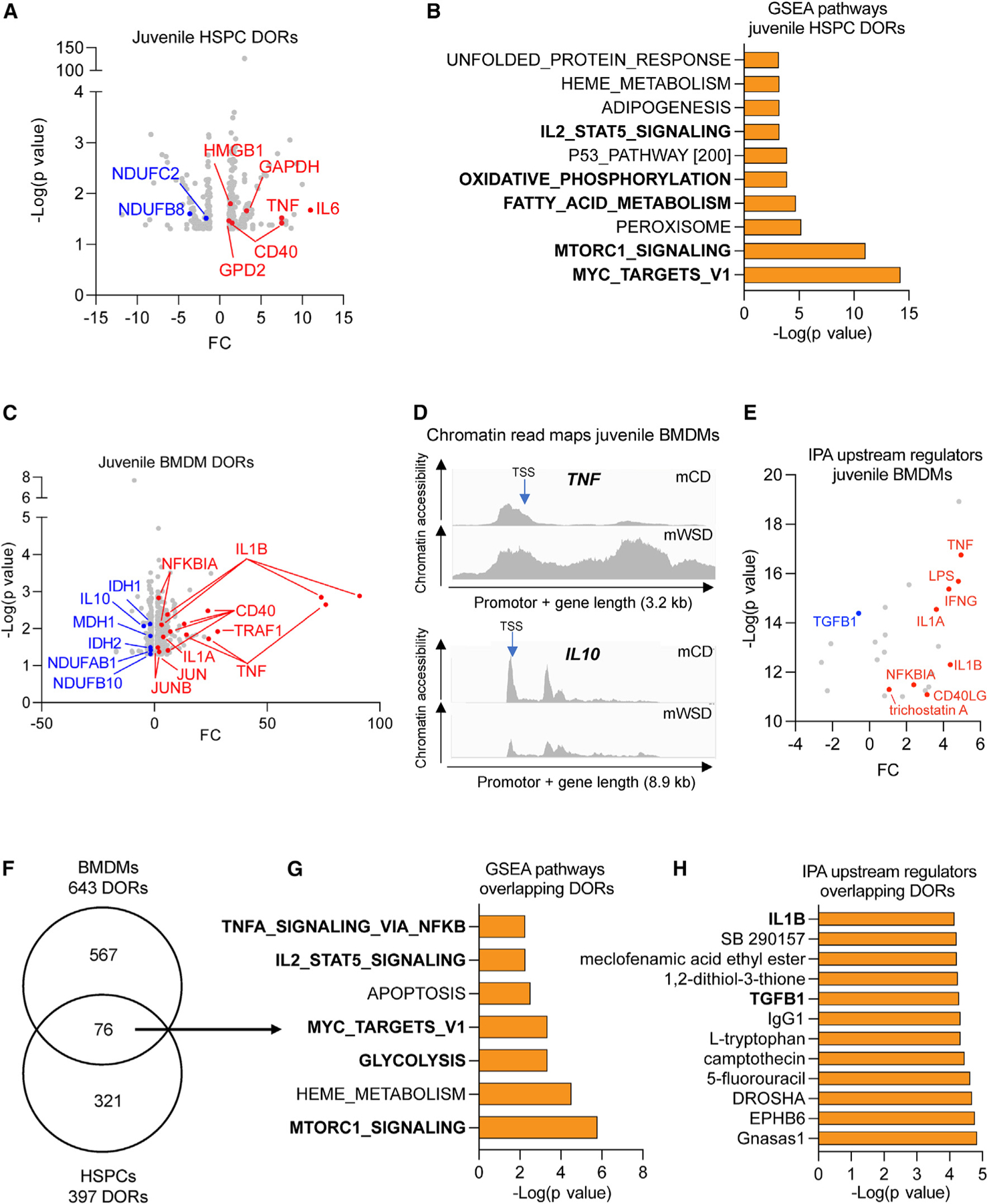
Exposure to mWSD is associated with differentially accessible chromatin architecture in juvenile offspring, with bias toward expression pathways regulating inflammation and oxidative phosphorylation (A) Volcano plot showing –Log(p value) and FC of DORs in mWSD- vs. mCD-exposed juvenile HSPCs. Key open (red) and closed (blue) loci are labeled. (B) GSEA of juvenile HSPC DORs. GSEA terms are shown, with select pathways bolded. (C) Volcano plot showing –Log(p value) and FC of DORs in mWSD- vs. mCD-exposed juvenile BMDMs. Key open (red) and closed (blue) loci are labeled. (D) Chromatin read maps of *TNF* and *IL10*. The y axis indicates ATAC-seq signal intensity (open chromatin). The entire gene body, including promotor region, for *TNF* and *IL10* is shown. Transcription start site (TSS) is denoted by blue arrow. Length of promotor and gene is shown in kilobases (kb). Average reads for n = 5 juvenile mCDs and n = 5 juvenile mWSDs are shown. (E) IPA upstream regulator analysis from juvenile BMDM DORs. Red indicates a positive *Z* score and upregulation. Blue indicates a negative *Z* score and downregulation. Other pathways (gray) are described in Table S7. (F) Venn diagram showing distinct and overlapping DORs in juvenile mWSD- vs. mCD-exposed HSPCs and BMDMs. (G) GSEA pathway analysis of overlapping DORs. GSEA terms are shown, with select pathways bolded. (H) IPA analysis of overlapping DORs, with select upstream regulators bolded. n = 5 juvenile mCDs, n = 5 juvenile mWSDs.

Induction of innate immune cell memory promotes glycolysis and inflammation in HSPCs and their myeloid progeny, suggesting that the metabolic features of BMDMs are inherited from hematopoietic precursors via epigenetic remodeling.^16,17,47^ To test whether mWSD exposure during gestation and lactation was associated with changes in chromatin accessibility that would support a heritable pro-inflammatory phenotype, we assessed chromatin accessibility via assay for transposase-accessible chromatin with sequencing (ATAC-seq) in promoter regions (± 1,000 bp around the transcription start site) of genes in HSPCs and BMDMs from mWSD- and mCD-exposed juveniles. In HSPCs from mWSD-exposed juveniles, we identified 179 differentially open gene regions (DORs) that had a negative fold change, indicating a less accessible chromatin state, and 381 loci that had a positive fold change, indicating a more accessible chromatin state (Figure 3A). Key DORs with increased accessibility mapped to the major pro-inflammatory cytokines *IL6* and *TNF*, as well as *CD40*, which is involved in antigen presentation and activation of inflammatory signaling pathways,^48^ and *HMGB1*, *GAPDH*, and *GPD2*, which regulate glycolysis (Figure 3A). Notable DORs with decreased accessibility included *NDUFB8* and *NDUFC2* (Figure 3A), which encode key components of the electron transport chain, and this is in line with reduced oxidative phosphorylation in these cells. Gene set enrichment analysis (GSEA) of mWSD-exposed HSPCs (Figure 3B) showed enrichment of inflammatory pathways (IL2/STAT5 signaling); MYC, a well-characterized factor that controls the balance of self-renewal and proliferation, as well as glycolytic activity, of HSPCs^49^; and enrichment of metabolic pathways such as MTORC1, oxidative phosphorylation, and fatty acid metabolism, supporting our metabolic findings in HSPCs.

Next, in mWSD-exposed BMDMs, we identified 470 DORs with less accessible chromatin and 537 DORs with more accessible chromatin (Figure 3C). Similar to HSPCs, DORs with increased accessibility in BMDMs from mWSD-exposed juveniles mapped to *IL1B*, *TNF*, *NFKBIA*, *IL1A*, *TRAF1*, and *CD40*, all major pro-inflammatory genes, as well as *JUN* and *JUNB*, which drive trained immunity in conjunction with *FOS*.^17^ Again, similar to mWSD-exposed HSPCs, DORs with reduced accessibility included *NDUFB10* and *NDUFAB1* (Figure 3C), which encode subunits of the mitochondrial electron transport chain, and *MDH1*, a key enzyme of the TCA cycle. Importantly, DORs with reduced accessibility also included *IL10*, an anti-inflammatory cytokine, as well as *IDH1* and *IDH2* (Figure 3C), encoding TCA cycle enzymes, which have decreased activity during the inflammatory response of macrophages, an effect expected to promote glycolysis.^50,51^ We discovered increased chromatin accessibility for *TNF* and *IL1B* at the promoter region and then extended the analysis of *TNF* and *IL1B* across the whole gene body in mWSD-exposed BMDMs, which showed increased chromatin accessibility as well (Figures 3D and S4, respectively). We also discovered decreased chromatin accessibility for *IL10* (Figure 3D), translating to both increased and decreased accessibility across broad regions of different individual genes. DORs in BMDMs were too numerous to analyze with GSEA, so we ran IPA analysis and identified upstream regulators (Figure 3E) promoting inflammation, including trichostatin A, a histone deacetylase inhibitor, as well as predicted downregulation of TGFB1, an anti-inflammatory regulator that also regulates HSC quiescence.^52^ Other pathways and upstream regulators predicted from DORs are shown in Tables S6 and S7.

To determine if the same genes were epigenetically regulated in progenitor (HSPC) and progeny (BMDM) cells in response to mWSD exposure, we compared the genes corresponding to DORs from the promoter regions and identified 76 genes with overlapping DORs (Figure 3F). Within this overlapping set of genes, using GSEA, we found enrichment of pathways TNFα/NF-κB signaling and IL2/STAT5 signaling from mWSD- vs. mCD-exposed cells, as well as metabolic pathways including glycolysis and MTORC1 in addition to the MYC pathway (Figure 3G). IPA upstream regulator analysis produced similar results with enrichment of *IL1B* and *TGFB1* in mWSD-exposed cells (Figure 3H). IPA pathways and other upstream regulators predicted from overlapping DORs are shown in Tables S8 and S9, respectively. Therefore, mWSD exposure is associated with overlapping epigenetic signatures supporting pro-inflammatory priming in both HSPCs and BMDMs from juveniles.

### mWSD upregulates genes associated with inflammation and initiation of immune memory in fetal bone marrow HSPCs

**Figure 4.**
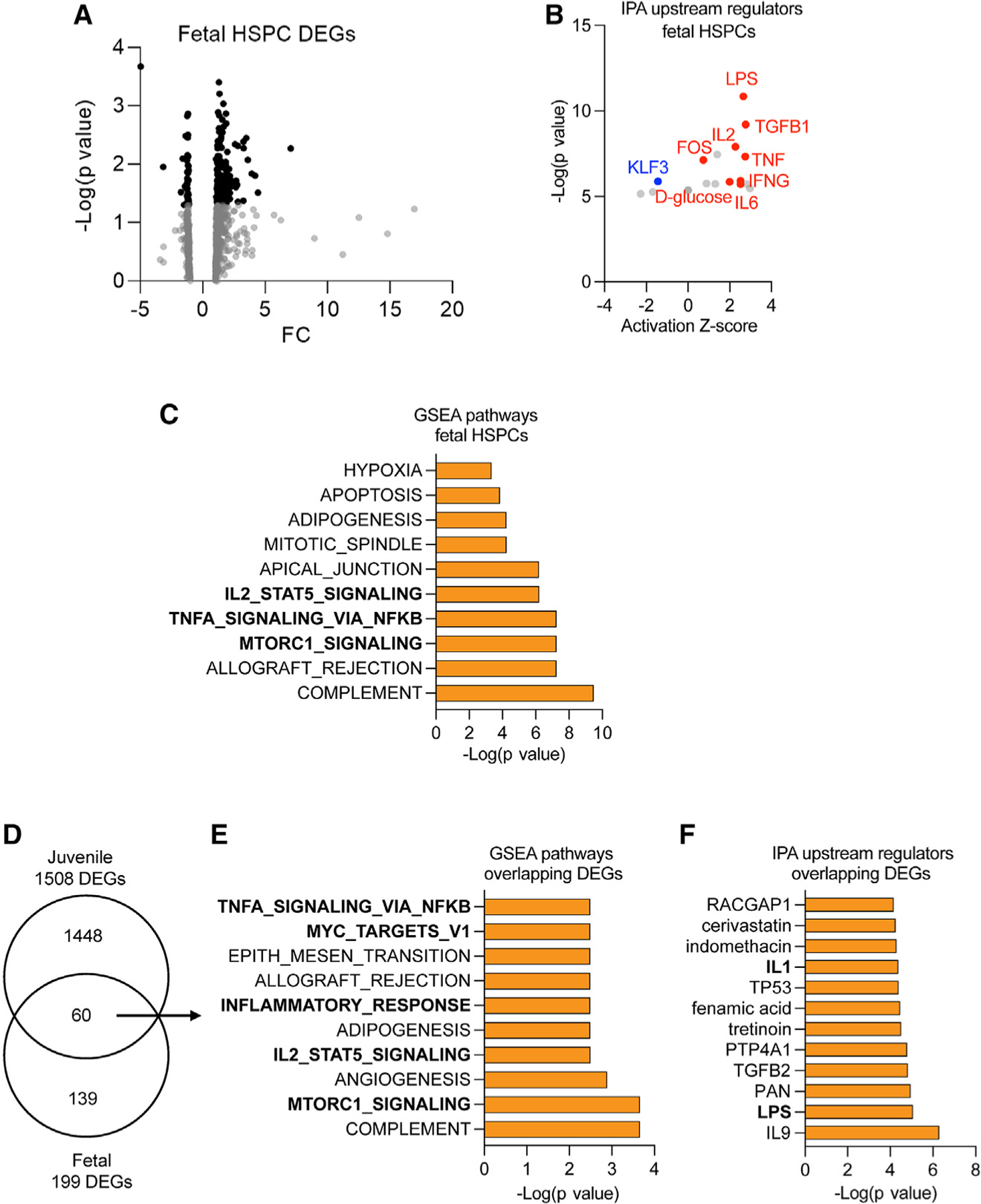
RNA-seq analysis of fetal bone marrow HSPCs (A) Volcano plot of –Log(p value) and FC of DEGs in mWSD- vs. mCD-exposed fetal HSPCs. Gray circles indicate genes with p > 0.05. Black circles indicate genes with p < 0.05 in mWSD vs. mCD, regardless of whether they passed quality check measures and thus regardless of whether they featured in the 199 DEGs used for subsequent analyses. (B) IPA upstream regulator analysis of DEGs in fetal HSPCs. Red indicates upstream regulators with a positive *Z* score and upregulation. Blue indicates upstream regulators with a negative *Z* score and downregulation. Other pathways (gray) are described in Table S11. (C) GSEA analysis of DEGs in fetal HSPCs. GSEA terms are shown, with select pathways bolded. (D) Venn diagram showing distinct and overlapping DEGs in fetal and juvenile bone marrow HSPCs. (E) GSEA pathway analysis of overlapping DEGs in HSPCs in mWSD-exposed fetuses and juveniles. GSEA terms are shown, with select pathways bolded. (F) IPA upstream regulator analysis of overlapping DEGs in HSPCs in mWSD-exposed fetuses and juveniles. Select upstream regulators are bolded. n = 5 fetal mCDs, n = 5 fetal mWSDs; n = 5 juvenile mCDs, n = 6 juvenile mWSDs.

We next asked whether gene signatures underlying pro-inflammatory priming in HSPCs from juveniles were present in HSPCs from early third-trimester fetuses, which would establish the intrauterine period as a critical window for pro-inflammatory priming of HSPCs.^10^ Thus, we compared gene expression in bone marrow HSPCs from mWSD- and mCD-exposed macaque fetuses using bulk RNA-seq. Like HSPCs from mWSD-exposed juveniles, PCA cleanly separated HSPCs from mWSD- and mCD-exposed fetuses (Figure S5). We identified 199 DEGs (p < 0.05), of which 155 were upregulated and 44 downregulated with mWSD exposure (Figure 4A). Like HSPCs and BMDMs from mWSD-exposed juveniles, IPA analysis showed upregulation of inflammatory upstream regulators, including LPS, TNF, IFNG, IL2, and IL6 (Figure 4B). Additional upregulated upstream regulators included FOS, which is important for the establishment and recall of innate immune memory.^17^ Consistent with increased glycolysis in HSPCs and BMDMs from mWSD-exposed juveniles, upregulation of genes involved in shuttling of glucose through glycolysis (D-glucose pathway) was found. KLF3, a mediator of erythroid differentiation in HSPCs,^53^ was downregulated, potentially reflective of reduced erythroid activity in HSPCs from mWSD-exposed juveniles (Figure 4B). Other pathways and upstream regulators predicted from DEGs in fetal HSPCs are shown in Tables S10 and S11, respectively. GSEA showed enrichment of TNFα/NF-κB signaling, IL2/STAT5 signaling, and MTORC1 signaling (Figure 4C).

Comparing the results from fetal and juvenile HSPCs, we identified 60 overlapping DEGs (Figure 4D). Within these DEGs, GSEA analysis showed enrichment of TNFα/NF-κB signaling, IL2/STAT5 signaling, and MYC and MTORC1 pathways (Figure 4E). Shared upstream regulators identified with IPA analysis included pro-inflammatory regulators LPS and IL1 (Figure 4F and Table S12).

To identify putative transcription factor interactions in DEGs from HSPCs from mWSD-exposed fetuses and juveniles, we used potentially interacting transcription factor finder (PC-TraFF) analysis.^54^ We found predicted interactions in mWSD-exposed fetal HSPCs between EGR, ETS, MAX, and MYC with SP, FOS with JUN, and C/EBPB with STAT6 (Table S13); some of these genes have been identified as pioneer factors promoting inflammation and immune memory.^16,17^ DEGs from HSPCs from mWSD-exposed juveniles were enriched for interactions between FOS with JUN, various C/EBP transcription factors with STAT6, ETS1 with NF-κB, and ETS1 with EGR1 (Table S13). These results suggest that increased pro-inflammatory gene expression and signatures of priming of inflammation in mWSD-exposed fetal HSPCs are also present in mWSD-exposed juvenile HSPCs.

### Metabolism and functional potential of mWSD-exposed fetal HSPCs mirrors juvenile HSPCs

**Figure 5.**
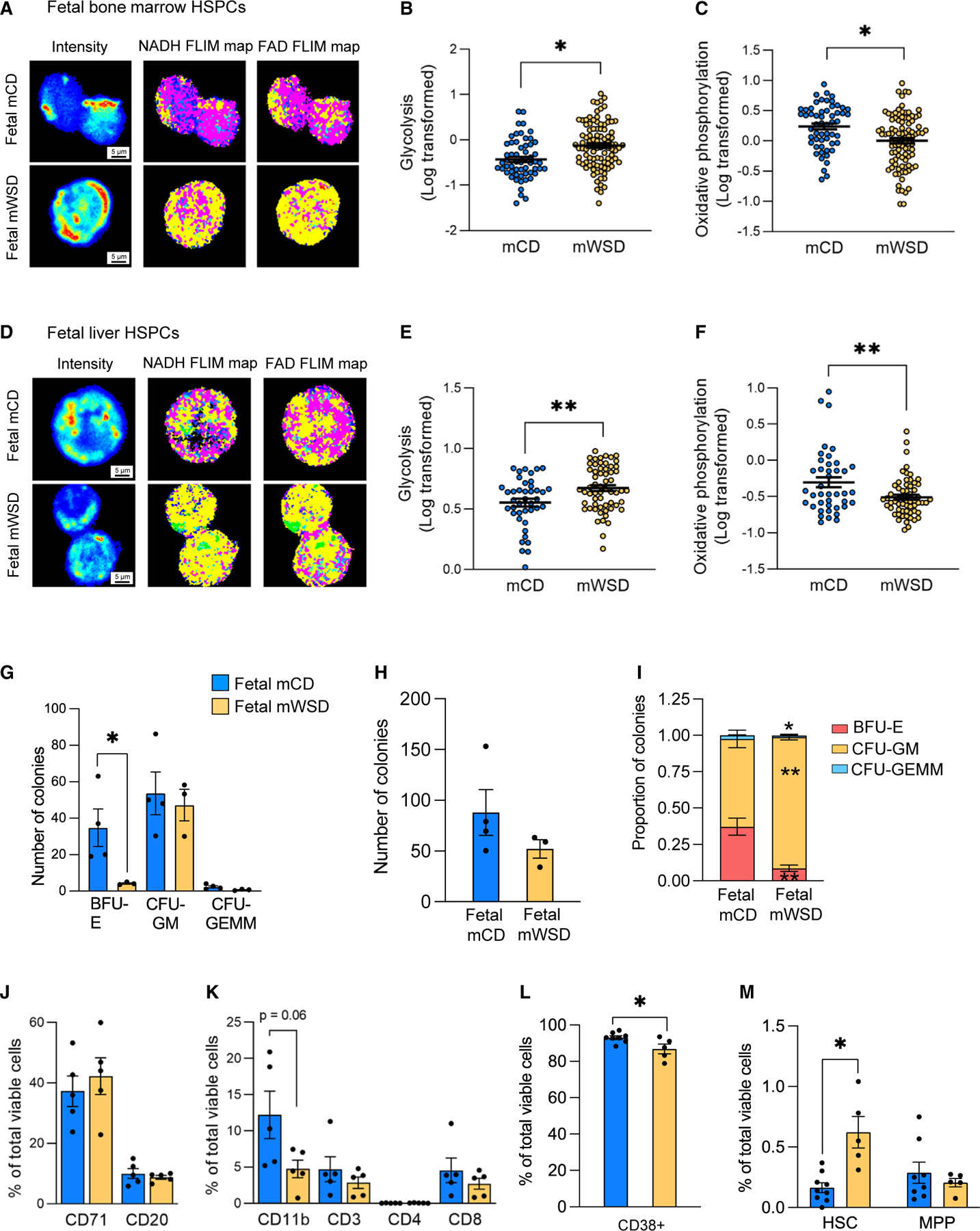
Analysis of metabolic phenotype of fetal HSPCs and composition of bone marrow (A–F) FLIM map of fetal HSPCs. FLIM map of fetal bone marrow HSPCs (A). For intensity in FLIM maps, red and yellow indicate higher intensity and blue and green indicate lower intensity of fluorescence lifetimes. For NADH maps, yellow indicates free NADH and pink indicates bound NADH. For FAD maps, yellow indicates bound FAD and pink indicates free FAD. Glycolytic index (B) and FLIRR (C) in mCD- and mWSD-exposed fetal bone marrow HSPCs. FLIM map of fetal liver HSPCs (D). Glycolytic index (E) and FLIRR (F) in mCD- and mWSD-exposed fetal liver HSPCs. (B, C, E, and F) Each dot represents one HSPC; 7–20 cells used per animal. (G–I) CFU assays of fetal bone marrow MNCs. Colony count from mCD- and mWSD-exposed fetal bone marrow MNCs (G). Total number of colonies grown from mCD and mWSD bone marrow MNCs (H). Proportion of CFU-GEMM, CFU-GM, and BFU-E colonies (I). Proportion of colonies was calculated by dividing the total count for each colony type per animal by the total colony number per animal. These calculations were input as n = 1. n = 4 mCDs, n = 3 mWSDs. (J–M) Flow cytometry analysis of fetal bone marrow MNCs. Fetal CD71+ cells and CD20+ B cells as a percentage of total viable cells (J). Overall CD11b+ myeloid cells and CD3+, CD4+, and CD8+ T cells as a percentage of total viable cells (K). CD38+ cells (L) and HSCs (CD34+CD38−CD45RA−CD90+) and MPPs (CD34+CD38−CD45RA−CD90−) (M) as a percentage of total viable CD34+ cells in mCD and mWSD. n = 5–9 mCDs, n = 5 mWSDs. Data are mean ± SEM (B, C, and E–M). *p < 0.05, **p < 0.01 mWSD effect from repeated measures ANOVA with log-transformed data (B, C, E, and F), unpaired Student’s t test (G–I), or Mann-Whitney U test (J–M).

Given similar gene signatures supporting inflammation and increased glycolysis in fetal and juvenile HSPCs, we again utilized FLIM to measure glycolysis and oxidative phosphorylation in HSPCs from fetal bone marrow and liver. mWSD-exposed fetal bone marrow HSPCs had a 40% increase in glycolysis and a 25% decrease in oxidative phosphorylation compared with mCD-exposed HSPCs (Figures 5A–5C). Fetal liver HSPCs exposed to mWSD had a similar phenotype, with a 25% increase in glycolysis and a 50% decrease in oxidative phosphorylation (Figures 5D–5F). To confirm whether mWSD imparted similar effects on the functional potential of the fetal bone marrow as it did in juvenile bone marrow, we performed CFU assays using fetal bone marrow MNCs. In fetal bone marrow MNCs, the number of BFU-E colonies was decreased nearly 8-fold by mWSD, whereas CFU-GM and CFU-GEMM colonies were unchanged (Figure 5G). While this difference did not translate into a statistically significant change in the overall number of colonies formed by mWSD-exposed fetal bone marrow MNCs compared with mCD-exposed MNCs (Figure 5H), the proportion of CFU-GM was significantly increased in bone marrow MNCs from mWSD-exposed fetuses (Figure 5I). Unlike mWSD-exposed juvenile bone marrow and despite the change in clonogenic potential, no change in the frequency of phenotypic CD71+ erythroid progenitors or CD20+ B cells was observed (Figure 5J). In mWSD-exposed fetal bone marrow MNCs, a trend toward decreased frequency of CD11b+ myeloid cells (p = 0.06) was found. Frequencies of CD3+, CD4+, and CD8+ T cells from mWSD-exposed fetal bone marrow MNCs were unchanged (Figure 5K). We also characterized the immature bone marrow CD34+ HSPC compartment in mWSD-exposed fetuses. Despite an overall decrease in CD34+CD38+ cells (Figure 5L), we found a nearly 5-fold increase in phenotypic HSCs but no changes in MPPs in mWSD-exposed fetal bone marrow HSPCs (Figure 5M), similar to mWSD-exposed juvenile HSPCs. We also measured CBCs in mCD- and mWSD-exposed fetuses and found no changes in any of the measurements between groups (Table S14), similar to juveniles. Taken as a whole, these findings support shifts in metabolism toward glycolysis and distribution of fetal HSPC populations due to mWSD exposure.

### mWSD is associated with metabolic shifts in fetal bone marrow and liver innate immune cells

**Figure 6.**
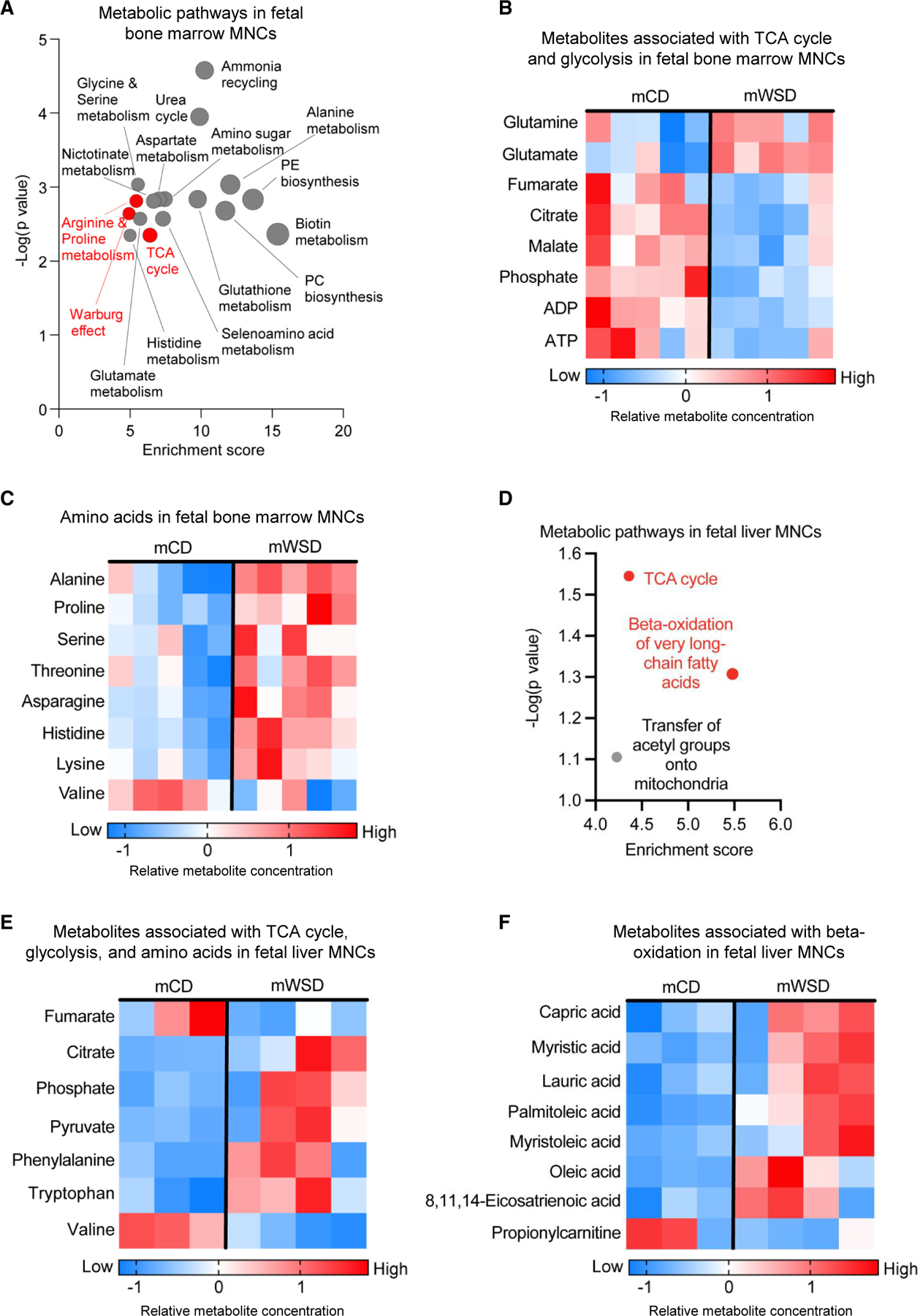
Fetal bone marrow and liver MNCs from WSD-fed dams are associated with altered TCA small molecular and metabolic intermediates (A) Enriched metabolic pathways from fetal bone marrow MNC top 25 VIP score metabolites. Red dots indicate pathways of interest and gray dots indicate other pathways. PE, phosphatidylethanolamine; PC, phosphatidylcholine. (B) Heatmap of metabolites associated with TCA cycle activity and glycolysis in mCD- vs. mWSD-exposed fetal bone marrow MNCs. (C) Heatmap of amino acids in mCD- vs. mWSD-exposed fetal bone marrow MNCs. (D) Enriched pathways from the top 25 VIP metabolites in fetal liver MNCs, red dots indicate pathways of interest, and gray dot indicates another pathway. (E) Heatmap of metabolites associated with TCA cycle activity, glycolysis, and amino acid metabolism in mCD- vs. mWSD-exposed fetal liver MNCs. (F) Heatmap of metabolites associated with β-oxidation in mCD- vs. mWSD-exposed fetal liver MNCs. One offspring per column (B, C, E, and F). n = 5 mCD, n = 5 mWSD bone marrow MNCs; n = 3 mCD, n = 4 mWSD liver MNCs.

To identify whether metabolic changes in fetal HSPCs were present in their innate immune progeny cells, we performed targeted metabolomics analyses on fetal bone marrow and liver MNCs using ultra-high-performance liquid chromatography-mass spectrometry (UHPLC-MS). PCA plots showed separation between mWSD and mCD groups in fetal bone marrow MNCs (Figure S6A) and liver MNCs (Figure S6B). Pathway analysis of the top 25 variable importance in projection (VIP) metabolites derived from fetal bone marrow MNCs (Figure S6C) showed enrichment for the TCA cycle and Warburg effect, as well as various amino acid metabolism pathways (Figure 6A), consistent with increased glycolysis and decreased oxidative phosphorylation. Further, the pathways associated with arginine metabolism, which accompanies a pathogenic M2 macrophage shift,^55^ were enriched in mWSD-exposed fetal bone marrow cells (Figure 6A). The abundance of malate, citrate, and fumarate was decreased (Figure 6B), potentially reflective of reduced TCA cycle activity. Phosphate metabolites were also decreased in mWSD-exposed fetal bone marrow MNCs (Figure S6C). Amino acid abundance was broadly increased following mWSD exposure (Figure 6C), suggesting either increased amino acid uptake or decreased catabolism of amino acids. In contrast, the branched-chain amino acid valine, indispensable for HSPC maintenance and proliferation,^56^ was decreased in mWSD-exposed fetal bone marrow MNCs (Figure 6C).

The top 25 VIP metabolites in mWSD-exposed fetal liver MNCs (Figure S6D) were enriched for pathways including the TCA cycle, β-oxidation of very-long-chain fatty acids, and transfer of acetyl groups onto mitochondria (Figure 6D). Pyruvate and citrate levels were increased, whereas fumarate was decreased (Figure 6E), potentially indicative of increased entry and removal of subsequent TCA cycle intermediates. mWSD-exposed fetal liver MNCs had increased free fatty acids (FFAs; oleic acid, capric acid, myristic acid, lauric acid, palmitoleic acid, and myristoleic acid), whereas propionylcarnitine, a metabolite of fatty acid oxidation, was decreased (Figure 6F). Collectively, these data indicate MNCs in mWSD-exposed fetal liver exhibit reduced TCA cycle activity in the setting of increased FFA accumulation.

### mWSD is associated with altered lipid profiles in fetal and juvenile hematopoietic tissues and increased adipocyte surface area in juvenile bone marrow

**Figure 7.**
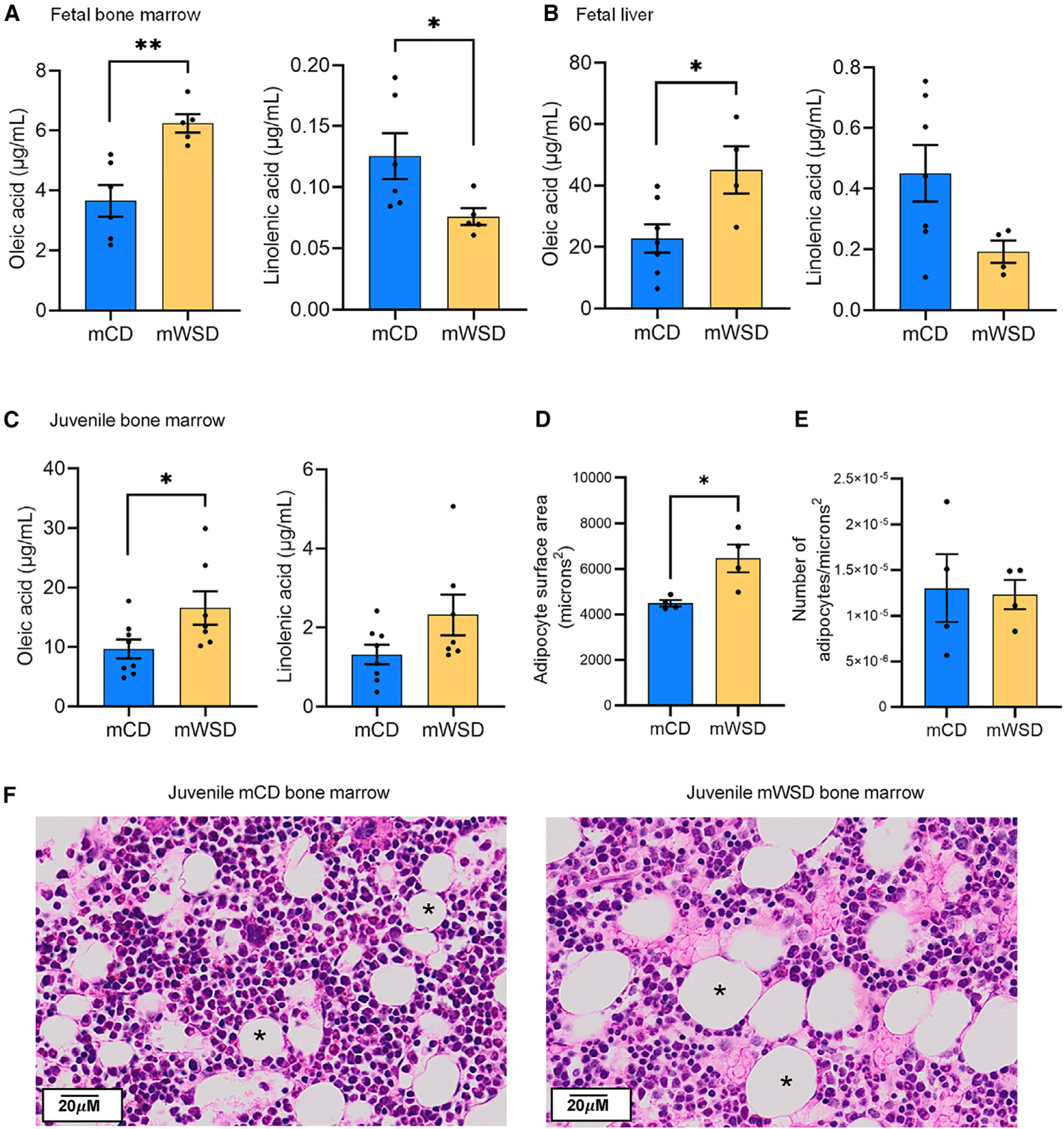
mWSD exposure is associated with increased oleic acid concentration in offspring bone marrow and liver (A) Concentration of oleic acid and linolenic acid in fetal bone marrow. (B) Concentration of oleic acid and linolenic acid in fetal liver. (C) Concentration of oleic acid and linolenic acid in juvenile bone marrow. (D) Mean cross-sectional surface area of adipocytes in juvenile bone marrow. (E) Adipocyte number per micrometer squared in juvenile bone marrow. (F) Representative images of juvenile bone marrow adipocytes. Asterisk (*) indicates adipocyte. Data are mean ± SEM. *p < 0.05, **p < 0.01, unpaired Student’s t test (A–E). n = 6–7 fetal mCDs, n = 4–5 fetal mWSDs; n = 8 juvenile mCDs, n = 7 juvenile mWSDs (A–C). n = 4 juvenile mCDs, n = 4 juvenile mWSDs (D and E).

Increased oxidation, synthesis, or uptake of lipids by hematopoietic cells can induce inflammatory myeloid cell production and can modulate HSPC self-renewal and differentiation.^57–60^ Particularly, uptake and oxidation of FFAs are essential for HSPC differentiation^59,61^ and loss of FFA oxidation leads to a complete loss of HSCs,^62^ implying a major role for FFAs in modulating HSPC function. To test whether mWSD exposure resulted in increased FFAs and eicosanoids in major sites of hematopoiesis, we measured the content of FFAs and eicosanoids in fetal liver and bone marrow and in juvenile bone marrow. In mWSD-exposed fetal bone marrow and liver, oleic acid was increased and linolenic acid was decreased (Figures 7A and 7B), with no other changes in FFA species (Table S15). In mWSD-exposed juvenile bone marrow, both oleic acid and linolenic acid were increased (Figure 7C), with no other changes in FFAs (Table S15). For eicosanoids in fetal bone marrow, prostaglandin E2 (PGE2) was decreased with mWSD exposure, as was the eicosanoid precursor arachidonic acid (AA) and docosahexaenoic acid (DHA) (Table S16). In mWSD-exposed fetal livers, eicosapentaenoic acid (EPA) was decreased, with no differences in other eicosanoid species (Table S16). In mWSD-exposed juvenile bone marrow, thromboxane B2 (TXB2) was decreased, with no other eicosanoid changes (Table S16). Last, because contact between bone marrow adipocytes and HSPCs can skew HSPC differentiation toward the myeloid lineage and produce inflammatory myeloid cells,^63,64^ we investigated adipocyte number and area in juvenile bone marrow. We found increased adipocyte mean cross-sectional area, but not abundance, in mWSD-exposed juvenile bone marrow (Figures 7D–7F). Thus, mWSD exposure increased oleic acid in fetal bone marrow and liver, and this increase persisted in the juvenile bone marrow, together with increased adipocyte cross-sectional area.

## DISCUSSION

We demonstrate that mWSD exposure is associated with *in utero* developmental programming of HSPCs to a pro-inflammatory phenotype that persists in HSPCs and BMDMs in 3-year-old juvenile offspring, 2.5 years after weaning to a control diet. BMDMs from mWSD-exposed juvenile offspring had a robust pro-inflammatory transcriptional and functional phenotype at baseline and when stimulated with TLR agonists. Further, mWSD exposure increased glycolysis and decreased oxidative phosphorylation in fetal HSPCs and immune cells in bone marrow and liver, and in juvenile bone marrow HSPCs and BMDMs. These data suggest that exposure to mWSD epigenetically modified inflammatory and metabolic genes in progenitor cells during fetal development, which promoted a pro-inflammatory phenotype that persisted after weaning to CD into juvenile life. This supports a model whereby HSPCs in juvenile animals act as a reservoir of parent cells with intrinsic epigenetic programming transmitted to myeloid cells with a pro-inflammatory phenotype with increased glycolysis that primes the offspring for inflammation across the lifespan.

Our findings demonstrate pro-inflammatory inheritance from HSPCs to BMDMs, associated with mWSD exposure, as shown by the transcriptomics, metabolomics, FLIM, and ATAC-seq data. Macrophage inflammatory responses can be regulated epigenetically, and enhanced accessibility of genes promoting glycolysis, inflammatory response, or antimicrobial responses in HSPCs can transmit to progeny cells such as BMDMs and affect their function.^65–67^ In juvenile HSPCs and BMDMs, mWSD exposure was associated with open chromatin around genes promoting glycolysis and less-open chromatin around genes promoting oxidative phosphorylation, indicating that a metabolic shift toward glycolysis is a key feature of mWSD-driven inflammatory priming. Notably, in earlier work using mWSD-exposed fetal HSPCs, we also found that mWSD led to poor engraftment of fetal HSPCs in nonlethally irradiated immunodeficient NOD/SCID/IL2rγ−/− mice.^68^ This suggests that alterations in the maternal environment may exert long-lasting effects on offspring immunity by reprogramming HSPCs in the fetal bone marrow and the fetal liver via intrinsic effects on HSPCs (e.g., changes in HSPC transcriptome).

Several factors associated with mWSD exposure may initiate metabolomic reprogramming in the fetal HSPCs. We found increased chromatin accessibility of gene regulatory regions and RNA signatures supporting activation of factors with a major role in regulating macrophage inflammatory activation including FOS/JUN, NF-κB, C/EBPβ, and STAT6 in juvenile BMDMs and HSPCs exposed to mWSD. Recent work has demonstrated that FOS/JUN^17^ and C/EBPβ^16^ are key for establishing and recalling inflammatory responses in progenitor and progeny cells. Our prior work also demonstrated a persistently altered histone code in liver tissue from juvenile animals exposed to mWSD during gestation and lactation,^9,29^ further supporting epigenetic programming in the fetal liver niche. In adult mice, recent data show that high-fat-diet-induced obesity triggered persistent epigenetic reprogramming in myeloid progenitor cells through stearic acid-induced *Il1b* expression.^26^ Likewise, in adult mice, exposure to IL-1bβ or β-glucan triggered trained immunity and increased glycolysis via HSPCs.^15,69^ Using an *in silico* analysis of RNA-seq and ATAC-seq from juvenile mWSD BMDMs, we found that IL-1B could be an important upstream regulator. However, there were no increases in pro-inflammatory cytokines in mWSD-exposed fetal serum or livers.^70^ Our previous work also showed that mWSD-exposed fetuses have increased hepatic oxidative stress, increased circulating lipid abundance, altered circulating lipid composition reflected by the WSD, and hypoxemia.^28,33,70^ The latter three represent systemic exposures that together could be expected to affect the HSPC niche during development in both the fetal liver and bone marrow.^71^ Specifically, these exposures associated with mWSD may shape the transcriptional and epigenetic landscape of HSPCs, thereby modulating HSPC differentiation, plasticity, and cellular identity.^63,72^ Given that hypoxia can promote glycolytic metabolism in both HSPCs^73^ and macrophages,^74^ we speculate that mWSD-induced fetal hypoxemia and oxidative stress *in utero* are early stimuli leading to local inflammatory transcriptional and metabolic phenotypes in HSPCs and BMDMs.

Surprisingly, although offspring were neither obese nor consumed a WSD in postnatal life, mWSD exposure during gestation and lactation increased adipocyte cross-sectional area in juvenile bone marrow, indicating a potential role for increased bone marrow adiposity in driving inflammatory priming in HSPCs. Bone marrow adipocytes in mice were initially described as negative regulators of hematopoiesis under regenerative stress.^75^ However, bone marrow adipocytes or FFAs can also have direct effects on HSPC differentiation, self-renewal, and may increase myeloid cell production.^63,76^ We found an increased concentration of oleic acid in mWSD-exposed fetal and juvenile hematopoietic tissues and fetal plasma.^28^ This is important because oleic acid can trigger production of inflammatory myeloid cells and potentiate HSPC expansion and increase HSPC proliferation *in vitro*.^58^ Further, oxidation of FFAs is key for HSPC differentiation and inflammatory response.^57,59^ Juvenile bone marrow (the site of central hematopoiesis postnatally) and fetal liver (the site of tissue resident macrophage production) exposed to mWSD had increased oleic acid and HSPCs, and MNCs from these tissues had evidence of increased glycolysis and decreased TCA cycle activity, consistent with sustained pro-inflammatory activation. Thus, maternal diet-supplied lipids, including oleic acid, in hematopoietic tissues may play an important role in priming inflammation and metabolism in fetal HSPCs and BMDMs with persistence postnatally.

Imprinting of the fetal immune system by the microbiota during the neonatal period has been demonstrated,^77^ and microbial products can alter hematopoiesis.^78^ We have shown that dysbiosis of the maternal gut microbiome in our WSD-fed Japanese macaque model persists in offspring 1 year after delivery,^30^ which could trigger epigenetic memory within offspring HSPCs.^79^ There are no reports directly linking the maternal transfer of dietary metabolites to neonatal hematopoietic and immune cells; however, dysbiosis in the mother could have indirect effects on the developing neonatal immune system either *in utero* or during lactation by altering microbial metabolites. Indeed, microorganisms are present in the placenta^80^ and in breast milk^81,82^ that play an important role in the development of the infant gut. mWSD exposure during lactation is known to contribute to development of metabolic diseases, particularly in rodent models.^83–86^ The striking changes in the fetal bone marrow and liver HSPCs observed here suggest that the primary driver for developmental programming of inflammation takes place *in utero*. However, we cannot rule out the possibility that exposure to mWSD during lactation triggers shifts in microbiome composition or function contributing to inflammation postnatally.

Previously, we demonstrated that diet reversal of obese dams to normal chow diet during pregnancy, or maternal supplementation of WSD with the antioxidant resveratrol, prevented oxidative stress and steatosis in mWSD-exposed fetal liver.^33,70,87^ Importantly, these results suggest that components of the maternal diet, rather than maternal obesity per se, are a modifiable risk factor with the potential to alter developmental programming of the immune system in offspring. Other studies have found that maternal glycemic index or blood lipids during obese pregnancy are associated with infant cord blood DNA methylation.^88–90^ Further, we have characterized histone modifications sensitive to mWSD exposure in our macaque model.^9,91,92^ Given that myeloid-biased reprogramming of HSPCs contribute to pro-inflammatory macrophages in adipose tissue and liver, and that HSPCs are a continuous source of progeny monocytes/macrophages throughout life, it will be important to determine causal mechanism.

Interestingly, while the 3-year-old juveniles studied here were not obese, we previously found in these same offspring a significant increase in hepatic periportal collagen deposition, a hallmark of pediatric nonalcoholic steatohepatitis, with transcriptional and metabolic pathways underlying hepatic oxidative stress, compromised mitochondrial lipid sensing, and decreased antioxidant response.^93^ Given that changes in mitochondrial bioenergetics in monocyte-derived macrophages are a critical determinant of fibrotic repair and resolution of tissue injury in nonalcoholic steatohepatitis and other diseases,^94,95^ we speculate that metabolically reprogrammed pro-inflammatory HSPCs play a critical role in either maladaptive repair or fibrosis underlying pediatric NAFLD.

### Limitations of the study

The metabolic flexibility and multiplicity of functions in tissue macrophages, including their roles in the initiation/resolution of inflammation, require further clarification in our model.^96^ The WSD-fed offspring studied here show evidence of liver fibrosis and pre-clinical NAFLD,^93^ suggesting macrophage polarization toward a non-restorative phenotype due to cellular factors within tissues that impair/activate this process. Spatial proteome and transcriptome studies with single-cell resolution need to be performed to explore the key processes and pathways specific to the bone marrow and different macrophage populations and regions in the liver or adipose tissue. Future studies can also confirm the contribution of oleic acid or IL-1β accumulation as a trigger for heritable pro-inflammatory priming of mWSD-exposed fetal HSPCs, and the extent to which other environmental triggers, such as hypoxemia or products from an altered maternal microbiome, participate in the process. In addition, epigenetic regulation can be transmitted to offspring through the paternal germline^97^; however, in our study, we analyzed the maternal but not paternal phenotype.

## STAR★METHODS

### RESOURCE AVAILABILITY

#### Lead contact

Further information and requests for resources and reagents should be directed to and will be fulfilled by the lead contact, Jed Friedman (jed-friedman@ouhsc.edu).

#### Materials availability

This study did not generate new unique reagents.

#### Data and code availability

- Bulk RNA-seq and ATAC-seq data have been deposited to GEO and are publicly available as of the date of publication. Accession number is listed in the key resources table.
- This paper does not report original code.
- Any additional information required to reanalyze the data reported in this paper is available from the lead contact upon request.

### EXPERIMENTAL MODEL AND SUBJECT DETAILS

#### Study approval

All animal procedures were conducted in accordance with the guidelines of the Institutional Animal Care and Use Committee (IACUC) of the Oregon National Primate Research Center (ONPRC). The ONPRC abides by the Animal Welfare Act and Regulations enforced by the United States Department of Agriculture.

#### Nonhuman primate models

For fetal studies, rhesus macaque females were fed CD or WSD for 5.5 years prior to pregnancy, as reported previously.^37,102,103^ The CD (Fiber-balanced monkey diet, no. 5000; Purina Mills, St. Louis, MO) contains 15% calories from fat, 27% from protein, and 58% from carbohydrates and the WSD (TAD primate diet, no. 5LOP, Purina Mills) contains 36% calories from fat, 18% from protein, and 46% from carbohydrates. At approximately 5.5 years of age, animals underwent timed-mated breeding and gestation day 130–135 fetuses (average gestational length is 165 days) were obtained via cesarean section. Maternal phenotypic measurements were collected as described previously.^102,103^ Fetuses were euthanized, weighed, and blood was drawn from the abdominal aorta post-pentobarbital for analysis of CBCs using a Horiba ABX Pentra 60C + Hematology analyzer (Kyoto, Japan). Femurs, tibias, and livers were removed during necropsy and shipped overnight in PBS (bones) or Belzer UW solution (liver; Bridge to Life, Northbrook, IL) on ice. Liver pieces were flash frozen and shipped overnight on dry ice for future lipid analyses.

For studies of juvenile offspring, Japanese macaque females were fed CD or WSD for 1–8 years prior to pregnancy, as reported previously.^3,27,104^ Sires were co-housed with dams in their respective maternal diet groups. The housing conditions and social status of the animals has been previously reported.^27,29,30,104^ Maternal phenotypic data collection included body fat composition, measured by dual-energy X-ray absorptiometry (DEXA; Hologic QDR Discovery A; Hologic, Inc., Bedford, MA), and body weight prior to pregnancy, and plasma insulin and glucose were obtained during pregnancy. IVGTTs were performed during the early third trimester of pregnancy. Juveniles were delivered naturally and kept with dams on their respective diets during lactation until weaning at 7–8 months of age, at which point all offspring were assigned to postweaning CD, regardless of maternal diet, and maintained on that diet until necropsy at 3 years of age. IVGTTs were performed within two months of necropsy as described.^32,34^ DEXA scans were performed to assess body composition within a month prior to necropsy.^32^ Juveniles were sacrificed and blood was drawn from the abdominal aorta post-pentobarbital for analysis of CBCs. During necropsy, sterna were collected for bone marrow adipocyte analyses and femurs and tibias were removed and shipped in PBS overnight on ice. Juvenile livers were studied previously,^93^ therefore were not included in the present study.

### METHOD DETAILS

#### Bone marrow MNC isolations

On shipment arrival, fetal and juvenile tibia and femur bones were cut on both ends and flushed with a total of 50 mL of D-PBS into a 50 mL conical tube. Bone marrow/D-PBS flushing was passed through a 100-micron cell strainer, aliquoted into 4–15 mL conical tubes, and centrifuged at 450 × g for 10 min at 4°C. The bone marrow supernatant was collected and frozen in aliquots for future lipid analyses. The pellets were resuspended in D-PBS and then subjected to a Ficoll Paque Plus (Sigma-Aldrich, St. Louis, MO) gradient in 4–15 mL conical tubes using an equal volume of cells/D-PBS to Ficoll, with Ficoll carefully pipetted into the centrifuge tube underneath the cells/D-PBS mixture. The gradient tubes were centrifuged at 450 × g for 10 min at 4°C with brake turned off (set to zero). The cell layer at the Ficoll/D-PBS interface was carefully removed and transferred to a new centrifuge tube. D-PBS was added to the tube to dilute any Ficoll carried over and centrifuged at 450 × g for 10 min at 4°C. The pellet was resuspended in RBC lysis buffer and incubated for 5 min on ice. An equal volume of D-PBS was added to end the reaction and the cells were centrifuged at 450 × g for 10 min at 4°C. The pellet was resuspended in D-PBS, counted, and the cells were allocated using two preservation methods. One aliquot of bone marrow MNCs was cryopreserved in 45% IMDM, 45% FBS, and 10% DMSO at a concentration of 20,000,000 cells per tube, frozen in a freezing container at −80°C, and transferred to liquid nitrogen for long term storage. A second aliquot of 2–5 million cells for metabolomics was transferred to a microfuge tube, centrifuged at 10,000 × g for 5 min at 4°C and the pellet was stored −80°C.

#### Liver MNC isolation

Liver MNCs were obtained via perfusion and collagenase digestion of a portion of the right lobe of the liver from CD and WSD fetuses.^24,70^ Hepatocytes were removed from the total mixture of digested cells by centrifugation at 100 × g for 5 min. The supernatant was filtered and spun at 800 × g for 10 min at 4°C to pellet nonparenchymal cells. Cells were resuspended in 24% Histodenz (Sigma) and gradients were prepared and spun at 1500 × g for 20 min at 4°C with acceleration set to one and brake turned off (set to zero). Cells at the interface (liver MNCs) were collected, washed, and aliquots were cryopreserved in 90% FBS/10% DMSO, stored at −80°C, and used for enrichment of liver HSPCs or frozen for metabolomics after RBC lysis (as described for bone marrow MNCs).

#### BMDM production

Cryopreserved bone marrow MNCs from juveniles were thawed and plated in 50 mL macrophage media (DMEM [1 g/L glucose] supplemented with 100 units/mL penicillin-streptomycin, 10% FBS, 100 units/mL MEM non-essential amino acid solution, and 25 ng/mL human M-CSF [Shenandoah Biotechnology, Warminster, PA]) for 10 days in T75 flasks. Media was changed on the third, fifth, and eighth day and BMDMs were harvested on day 10.

#### BMDM NanoString gene expression analysis

BMDMs from juveniles were grown for 10 days, at which point they were plated onto a 6-well plate at a density of 500,000 cells per well in macrophage media. Cells adhered overnight and were then treated with 100 ng/mL LPS, 100 ng/mL LPS +10 ng/mL IFNγ, 100 ng/mL IL-4, or no treatment in fresh media for 4 h. Cells were then scraped, lysed, and RNA was isolated using an RNeasy mini kit (Qiagen, Germantown, MD). RNA was run on a nonhuman primate-specific nCounter NHP Immunology Panel (NanoString Technologies, Seattle, WA) at the University of Oklahoma Health Sciences Center Genomics Core Facility. Differentially regulated genes were defined as having an uncorrected p value less than 0.05, with a linear fold change of greater than or less than two.

#### Phagocytosis assay

After 10 days of growth/differentiation, BMDMs were seeded in macrophage media at a density of 50,000 cells per well in a black-walled, clear/flat bottom 96-well plate (Greiner Bio-One, Monroe, NC) and allowed to adhere for 3 h at 37°C with 5% CO_2_. Media was then removed and LPS was added at 100 ng/mL in macrophage media and cells were incubated for 20 h. pHrodo Red *E*. *coli* bioparticles (Thermo Fisher Scientific, Waltham, MA) were resuspended in Live Cell Imaging Solution (Thermo) and diluted to a concentration of 0.25 mg/mL. Treatment media was removed and replaced with 100 μL of diluted bioparticle solution and the plates were transferred into the BioTek Cytation 5 cell imaging multi-mode reader (Agilent, Santa Clara, CA) set at 37°C and the gas controller set at 5% CO_2_. One image per well from two technical replicates was taken every 10 min for 1 h using a 4× objective lens with brightfield and RFP filter cube. After the live cell imaging assay, an end-point assay was run using a 20× objective lens. Fluorescence signal was analyzed using Gen5 Image Prime software (Agilent) and quantified after applying a mask to exclude background noise. After the phagocytosis assay, protein amount was quantified using sulforhodamine B normalization (adapted from^105^ and^106^). Briefly, bioparticles were washed out with 1× PBS, cold 10% trichloroacetic acid was added, and the plate was incubated 1h at 4°C. After washing with water and air drying, 0.1% sulforhodamine B in 1% acetic acid was added and incubated for 30 min at room temperature for normalization of protein content per well. The plate was then washed with 1% acetic acid to remove excess sulforhodamine B, air-dried, and Tris base was added to solubilize protein-bound dye. The plate was read at 490 nm in the Cytation 5 and results were used to normalize the fluorescence signal.

#### Bone marrow and liver HSPC enrichment

HSPCs (CD34+ cells) were isolated from cryopreserved fetal or juvenile bone marrow MNCs or fetal liver MNCs via a MACS magnetic enrichment column (Miltenyi, Gaithersburg, MD). At least 10,000,000 viable bone marrow MNCs or 2,000,000 liver MNCs were incubated with anti-human CD34 antibody (BD Biosciences, clone 563) and human Fc block (Miltenyi) together using 3 μL and 6 μL, respectively, per 1,000,000 cells for 20 min at room temperature. The rest of the MACS column protocol was followed according to manufacturer’s instructions (Miltenyi). The CD34-flow-through fraction was collected in addition to the CD34+ fraction. CD34 enrichment efficiency was validated with flow cytometry. Enriched fractions yielded over 90% HSPCs.

#### Colony forming unit assays

Bone marrow MNCs were thawed and plated at 10,000 viable cells per 35 mm grid plate in 1 mL human methylcellulose complete media (R&D Systems, Minneapolis, MN) supplemented with 100 units/mL of penicillin-streptomycin. Plates were kept at 37°C for 7 days, then manually counted for colony number and type on a brightfield microscope. Cell morphology was used to determine colony type. Cell colonies were defined as having at least 50 cells and were classified as either burst forming unit-erythroid (BFU-E), colony forming unit-granulocyte/monocyte (CFU-GM), or colony forming unit-granulocyte/monocyte/megakaryocyte/erythrocyte (CFU-GEMM). Counts were done in triplicate.

#### Bulk RNA sequencing

RNA from HSPCs enriched from cryopreserved bone marrow MNCs was isolated using a Quick-DNA/RNA microprep kit (Zymo Research, Irvine, CA). RNA integrity values and concentration were obtained using a Tapestation system (Agilent). PolyA-enriched RNAs were sequenced as 2×150 bp reads on the NovaSeq 6000 platform (Illumina, San Diego, CA) at the University of Oklahoma Health Sciences Center Genomics Core Facility. Derived sequences were analyzed by applying a custom computational pipeline consisting of the open-source GSNAP, Cufflinks, and R for sequence alignment and ascertainment of DEGs.^107^ Reads generated were mapped to the rhesus macaque genome (mmul10) by GSNAP,^98^ expression (FPKM) derived by Cufflinks,^99^ and differential expression analyzed with ANOVA in R. DEGs (p < 0.05 and Q < 0.1) were generated by comparing each group of mWSD-exposed animals to mCD-exposed animals. Genes included in the final list of DEGs, which were analyzed through the methods outlined below, were subject to the following additional requirements. The mCD mean, mWSD mean, or absolute value of the mCD mean minus mWSD mean had to be greater than or equal to 5 FPKM. The list of DEGs (p < 0.05) in mWSD-exposed fetal HSPCs and in mWSD-exposed juvenile HSPCs were analyzed with the free online potentially collaborating transcription factor finder (PC-TraFF) tool.^54^ The human hg19 genome was used as the reference database. The upstream region was set to 1000 bp, downstream was set to 0 bp, the minimal distance between pairs was 5, the maximum distance between pairs was 20, and the *Z* score cutoff was 4.5.

#### Gene set enrichment analysis (GSEA)

For GSEA analysis, we utilized the molecular signatures database (MSigDB) version 7.5.1.^108^ We input lists of DEGs generated from RNA-seq or ATAC-seq analysis and selected “human” as the species. We then queried the hallmark gene set database. GSEA allowed for a maximum of 500 inputs. We filtered pathway hits to being greater than −log(p value) = 2.25.

#### GO analysis

Gene ontology (GO) analysis was performed using DAVID v2022q1.^109^ Genes were classified according to official gene ID and homo sapiens was used as the species. Pathways were generated by selecting “biological process” under “functional annotations”. Pathways with a p value <0.05 (−log(p) > 2.3) were reported.

#### Ingenuity pathway analysis of bulk RNA-seq and NanoString data

IPA software (Qiagen) was used in order to identify upstream regulators and canonical pathways enriched in DEGs generated from bulk RNA-seq analyses. Upstream regulators and canonical pathways each had a corresponding *Z* score, calculated by IPA, indicating predicted activation of the pathway (positive *Z* score), inactivation of the pathway (negative *Z* score), or neither activation nor inactivation of the pathway (*Z* score = 0). To analyze HSPC RNA expression, lists of DEGs (uncorrected p < 0.05) and their fold changes were input into IPA. One list corresponded to DEGs in mWSD-vs. mCD-exposed fetal bone marrow HSPCs and one corresponded to mWSD-vs. mCD-exposed juvenile bone marrow HSPCs. Due to the varying number of DEGs in HSPCs from fetuses and juvenile offspring, different cutoffs were applied to determine the significance for canonical pathways and upstream regulators. For fetal canonical pathways, pathways with a −log(p value) of 2.5 or higher were included and for juvenile canonical pathways, a cutoff of −log(p value) of 5.5 or higher was applied. For upstream regulators, a p value cutoff of 1E-6 or lower for fetuses and a −log(p value) of 6.5 or higher for juvenile offspring was applied. For analysis of the 60 overlapping DEGs in mWSD-exposed fetal and juvenile offspring HSPCs, a cutoff of p = 1E-5 or lower was applied and canonical pathways were not reported.

For BMDM gene expression in response to LPS + IFNγ or LPS, the expression of a large subset of DEGs was analyzed from the NanoString panel that were commonly differentially expressed (uncorrected p < 0.05) compared with each respective baseline (uncorrected p < 0.05) in both mCD and mWSD groups. These common DEGs and fold change vs. baseline were input into IPA to generate a list of predicted upstream regulators, using lists derived from BMDMs treated with LPS + IFNγ and from BMDMs treated with LPS. Due to the number of predicted upstream regulators, only regulators with a p value of less than 1E-75 for LPS + IFNγ and for LPS were reported, regardless of *Z* score of activation. To analyze gene expression in response to IL-4, upstream regulators from the full set of DEGs in mCD-exposed BMDMs in response to IL-4 were generated, regardless of expression in mWSD-exposed BMDMs. Upstream regulators were filtered from this set to have a p value of less than or equal to 1E-40. The same analysis for the gene set from mWSD-exposed BMDMs in response to IL-4 was performed, and IPA was used to generate a list of upstream regulators and corresponding Z-scores. Using these two lists, Z-scores of the same upstream regulators identified as p = <1E-40 in mCD-exposed BMDMs vs. the same upstream regulators in mWSD-exposed BMDMs (regardless of p value in mWSD group) were compared to assess if they were activated to a similar degree between mCD and mWSD groups.

#### Metabolomic analysis

Between 1,200,000 and 8,000,000 viable bone marrow MNCs and between 300,000 and 800,000 viable liver MNCs were used for metabolomics analyses. Cells were extracted in ice-cold lysis/extraction buffer (5:3:2 methanol:acetonitrile:water). Metabolite analysis was performed utilizing UHPLC-MS on a Vanquish UHPLC coupled online to a Q Exactive high resolution mass spectrometer (Thermo).^33^ Samples were resolved over a Kinetex C18 column (2.1 × 150 mm, 1.7 μm; Phenomenex, Torrance, CA) at 25°C using a 3 min isocratic condition of 5% acetonitrile, 95% water, and 0.1% formic acid flowing at 250 μL/min, or using a 9 min gradient at 400 μL/min from 5 to 95% B (A: water/0.1% formic acid; B: acetonitrile/0.1% formic acid). Metabolites with values of zero at greater frequencies than 50% of all values across diet groups were removed from further analysis. In cases where zero values made up less than 50% of all values, zeroes were replaced with ½ the value of the minimum reading across all samples for that metabolite. MetaboAnalyst 5.0 software was used for analysis of metabolic pathway enrichment. For bone marrow MNCs, data was normalized by sum with auto scaling, and for liver MNCs, data was normalized by median with auto scaling. Analysis was done in parallel for both cell types. In both bone marrow and liver MNCs, 130 metabolites were detected, with four more (5-phospho-alpha-D-ribose 1-diphosphate, (R)-S-lactoylglutathione, taurocholate, taurochenodeoxycholate) only detectable in liver MNCs. To analyze differences between mWSD and mCD groups, multivariate PCA was performed, and partial least squares discriminant analysis (PLS-DA) was used to identify the 25 metabolites with the highest VIP scores. These top 25 metabolites were input into a Pearson-Ward unsupervised heatmap generator in MetaboAnalyst. The lists of the top 25 VIP metabolites were also input into enrichment analysis through MetaboAnalyst, with a hypergeometric test, utilizing KEGG IDs to identify the compounds, and were mapped to the SMPDB database.

#### Immunophenotypic analysis of bone marrow cells

Cryopreserved bone marrow MNCs were thawed and viable cells were counted. One million viable cells were stained with DAPI viability dye, anti-human CD71 (BD Biosciences, clone L01.1), anti-human CD3 (BD Biosciences, clone SP34–2), anti-human CD4 (BD Biosciences, clone L200), anti-CD8b (Thermo, clone 3B5), anti-CD34 (BD Biosciences, clone 563), anti-human CD20 (BioLegend, clone 2H7), anti-CD14 (Miltenyi, clone TUK4), and anti-human CD11b (BD Biosciences, clone ICRF44) for 20 min at room temperature with Human Fc Block (Miltenyi). Alternatively, 200,000 CD34-enriched viable cryopreserved cells were thawed and stained with anti-human CD38 (Caprico Biotechnologies, clone OKT10), CD45RA (BD Biosciences, clone 5H9), CD90 (BD Biosciences, clone 5E10), and DAPI viability dye. Cells were then passed through a 100-micron cell strainer into FACS sorting buffer, run on a FACSAria flow cytometer (BD Biosciences), and analyzed using FlowJo software (BD Biosciences). Compensation controls were run with UltraComp eBeads (Thermo) during each run and fluorescence minus one (FMO) controls were run for each fluorophore in the panel. MPPs were designated as CD34+CD38-CD90-cells and HSCs were designated as CD34+CD38-CD90+ cells, as published previously in macaques.^110^

#### Fluorescence lifetime imaging microscopy (FLIM)

HSPCs (30,000 CD34+ cells) and 50,000 CD34-cells were plated onto a 35 mm high μ-dish (Ibidi, Gräfelfing, Germany) in duplicate in DMEM (1 g/L glucose) supplemented with 20% FBS and 100 units/mL penicillin-streptomycin. Cells adhered for 24 h, media was changed on the second day, and cells were imaged on the third day with media changed to Ringer’s solution (122.5 mM NaCl, 5.4 mM KCl, 1.2 mM CaCl_2_, 0.8 mM MgCl_2_, 0.8 mM Na_2_HPO_4_, 0.2 mM NaH_2_PO_4_, 5.5 mM glucose, and 10 mM HEPES, pH 7.4) immediately prior to imaging. For BMDM FLIM, bone marrow MNCs were grown into BMDMs in 35 mm high μ-dishes (Ibidi) in macrophage media with M-CSF for 10 days, using three dishes per animal. BMDMs were then treated with basal media, LPS, or IL-4 for 4 h, then media was removed and replaced with Ringer’s solution immediately prior to imaging.

FLIM was performed to detect local metabolic changes in 7–20 individual HSPCs/animal or 20–30 BMDMs/animal using a Zeiss 780 laser-scanning confocal/multiphoton-excitation fluorescence microscope with a 34-channel GaAsP QUASAR detection unit and non-descanned detectors for two-photon fluorescence (Zeiss, Thornwood, NY) equipped with an ISS A320 FastFLIM box and a titanium:sapphire Chameleon Ultra II (Coherent, Santa Clara, CA).^111,112^ Each cell was counted as n = 1. For the acquisition of FLIM images, fluorescence for the reduced form of nicotinamide adenine dinucleotide (NADH) and flavin adenine dinucleotide (FAD) was detected simultaneously by two photon-counting photomultiplier tube detectors (H7422p-40; Hamamatsu Photonics, Hamamatsu, Japan). Images of the cells were obtained with VistaVision software (ISS, Champaign, IL) in 256 × 256 format with a pixel dwell time of 6.3 μs/pixel and averaging over 30 frames. The number of pixels covered with lifetimes for free and bound NADH and FAD were calculated in SimFCS software (Laboratory for Fluorescence Dynamics, University of California, Irvine, CA) and the values were normalized to the total number of pixels detected.^111,112^ The glycolytic index was calculated for all experimental groups using the following equation: glycolytic index = free NADH fraction/bound to enzyme NADH fraction.^44,112^ To assess mitochondrial activity (oxidative phosphorylation), FLIM based optical redox ratio (fluorescent lifetime redox ratio = FLIRR) was calculated as follows: FLIRR = bound to enzyme NADH fraction/bound to enzyme FAD fraction.^44,112^

#### ATAC-seq

BMDMs and cryopreserved HSPCs (between 8,000 and 40,000 cells) were used for DNA isolation with an ATAC-seq kit (Active Motif, Carlsbad, CA). DNA integrity was assessed with NanoDrop (Thermo) and DNA was considered pure if it achieved a 260/280 ratio of 1.5 or higher. The ATAC template libraries were sequenced as 2×150 bp reads on the NovaSeq 6000 platform at the University of Oklahoma Health Sciences Center Genomics Core Facility. Derived sequences were analyzed using the PEPATAC pipeline.^113^ Within the PEPATAC pipeline, reads generated were mapped to the rhesus macaque genome (mmul10) by Bowtie2,^100^ with ATAC loci called using MACS2.^101^ Differential chromatin availability was analyzed with ANOVA in R. Differentially expressed peaks (p < 0.05) within 1000 bp of transcription start sites (TSS) of DEGs from the RNA-seq were compared using ANOVA in R. Hits in each list were defined as p < 0.05. Regions were mapped to the respective gene, and gene symbols were used to generate differentially open region (DOR) lists. All regions and fold change were input as corresponding to the respective associated gene symbol. Therefore, it was possible for DOR lists to have multiple values corresponding to different regions associated with the same gene symbol, and duplicates were not removed for our lists.

#### Ingenuity pathway analysis of ATAC-seq data

DORs from mWSD-vs. mCD-exposed juvenile HSPCs were analyzed, DORs from mWSD-vs. mCD-exposed juvenile BMDMs were analyzed, and DORs present in both mWSD-exposed HSPCs and BMDMs were analyzed. Loci were mapped to each corresponding gene symbol and each gene symbol and fold change of its loci were input into IPA. For genes with more than one differentially expressed loci, IPA automatically determined the highest magnitude of fold change for the gene and used that single gene input and corresponding fold change for pathway analysis. Therefore, multiple DORs per gene did not translate to input of the same gene into IPA analysis multiple times. Z-scores and p values were calculated for canonical pathways and upstream regulators in both comparisons by IPA software. For HSPC and BMDM canonical pathways, a cutoff of higher than −log(p) = 3 and −log(p) = 4 was chosen, respectively. For HSPC upstream regulators, due to a lower number of DORs used as input and more extraneous hits in IPA, regulators were filtered to p < 5E-6. For BMDM upstream regulators, a cutoff of p < 5E-12 was selected. For DORs overlapping between mWSD-exposed HSPCs and BMDMs, a cutoff of −log(p) = 1.7 was selected for canonical pathways and p < 5E-5 was selected for upstream regulators.

#### Free fatty acid/eicosanoid analysis

Free fatty acid analysis was performed by gas chromatography-mass spectrometry (GC/MS).^114^ The sample was acidified with HCl (70 mM final concentration), and stable isotope-labeled fatty acid standards [^13^C_4_]palmitic, [d_3_]stearic, [d_2_]oleic, and [d_8_]arachidonic acid were added. After vortexing, samples were extracted with 1 mL of isooctane. The extract was dried under N_2_ and derivatized by the addition of 25 mL each of 1% pentafluorobenzyl bromide and 1% diisopropylethylamine. The vials were incubated at room temperature for 20 min, dried under N_2_, and reconstituted in 100 mL of isooctane. Analysis of the samples was performed by negative ion chemical ionization GC/MS on a Finnigan DSQ GC/MS system (Thermo Finnigan, Thousand Oaks, CA). The mass spectrometer was operated in the negative ion chemical ionization mode using methane as reagent gas. Data were acquired by selected ion monitoring of the following fatty acids: lauric (m/z 199), myristic (m/z 227), palmitic (m/z 255), stearic (m/z 283), linolenic (m/z 277), linoleic (m/z 279), oleic (m/z 281), eicosapentaenoic (m/z 301), arachidonic (m/z 303), and docosahexaenoic acid (m/z 327). The ions at m/z 259, 286, 283, and 311 were monitored for [^13^C_4_]palmitic, [d_3_]stearic, [d_2_]oleic, and [d_8_]arachidonic acids, respectively. Concentration was determined using stable isotope dilution with standard curves generated for each free fatty acid.

#### Adipocyte imaging

Juvenile sterna were fixed in 4% PFA for 24 h at 4°C, decalcified in 0.5 M EDTA for 4–7 days, and embedded in paraffin. Adjacent 5-mm paraffin sections were stained with hematoxylin and eosin to evaluate adipocyte morphology. Whole slide scan images were captured using the Leica AT2 System (Leica Biosystems, Deer Park, IL). Bone marrow adipocytes were outlined manually and the mean cross-sectional adipocyte area and the adipocyte number per sternal bone marrow area were calculated using ImageJ software.

### QUANTIFICATION AND STATISTICAL ANALYSIS

Data were analyzed by unpaired two-tailed Student’s t test or by unpaired two-tailed Mann-Whitney test with Grubbs test for outliers using Graphpad Prism (version 9). The statistical test used is included in the figure legends and table footnotes. Metabolic phenotypes and blood cell count readings were analyzed by ANOVA with fixed effects of maternal diet (mWSD, mCD), sex, and interaction (SAS software, version 9.4). Sex effects in fetuses and juveniles were evaluated using a two-way ANOVA with fixed effects of diet and offspring sex. No significant effects of sex were found and because of the small sample size for some variables, sex was not included in the final analyses and data from female and male offspring were combined. For FLIM analyses, data was log-transformed and a repeated measures ANOVA with a fixed effect of maternal diet, or fixed effect of treatment, maternal diet, and interaction was used to determine significance. For phagocytosis, a repeated measures ANOVA with a fixed effect of treatment, maternal diet, and interaction was used to determine significance.

## Supplementary Material

1

## Figures and Tables

**Table:** KEY RESOURCES TABLE

REAGENT or RESOURCE	SOURCE	IDENTIFIER
Antibodies		

CD71 (L01.1) APC	BD Biosciences	Cat#341028; RRID: AB_400560
CD3 (SP34–2) BV650	BD Biosciences	Cat#563916; RRID: AB_2738486
CD4 (L200) BV605	BD Biosciences	Cat#562843; RRID: AB_2737833
CD8b (3B5) PE-Cy5.5	Thermo Fisher Scientific	Cat#MHCD0818; RRID: AB_1484921
CD34 (563) PE	BD Biosciences	Cat#550761; RRID: AB_393871
CD20 (2H7) BV510	BioLegend	Cat#302339; RRID: AB_2561721
CD14 (TÜK4) PE-Vio770	Miltenyi Biotec	Cat#130–113–149; RRID: AB_2725977
CD11b (ICRF44) PE	BD Biosciences	Cat#555388; RRID: AB_395789
CD38 (OKT10) APC	Caprico Biotechnologies	Cat#100844
CD45RA (5H9) BV711	BD Biosciences	Cat#740806; RRID: AB_2740469
CD90 (5E10) PE-Cy7	BD Biosciences	Cat#561558; RRID: AB_10714644

Chemicals, peptides, and recombinant proteins		

Human M-CSF	Shenandoah Biotechnology	Cat#100–03
LPS	Sigma Aldrich	Cat#L5418
IFNγ	Novus	Cat#NBP2–61327
IL-4	Novus	Cat#NBP2–35309
FcR Blocking Reagent, human	Miltenyi Biotec	Cat#130–059–901
UltraComp eBeads compensation beads	Thermo Fisher Scientific	Cat#01222242
Belzer UW solution	Bridge to Life Ltd.	Belzer UW
Ficoll Paque Plus	Sigma Aldrich	Cat#GE17–1440–02
Histodenz	Sigma Aldrich	Cat#D2158
Human methylcellulose complete media	R&D Systems	Cat#HSC003
pHrodo Red E. coli Bioparticles	Thermo Fisher Scientific	Cat#P35361
Live Cell Imaging Solution	Thermo Fisher Scientific	Cat#A14291DJ
Sulforhodamine B	Sigma Aldrich	Cat#230162

Critical commercial assays		

nCounter NHP Immunology Panel	NanoString	Cat#XT–CSO–NHPIM1–12

Deposited data		

Fetal HSPC bulk RNA-seq	This paper	Database: GSE219095
Juvenile HSPC bulk RNA-seq	This paper	Database: GSE219095
Juvenile HSPC ATAC-seq	This paper	Database: GSE219095
Juvenile BMDM ATAC-seq	This paper	Database: GSE219095

Experimental models: Organisms/strains		

Primate: Japanese macaque	ONPRC	N/A
Primate: rhesus macaque	ONPRC	N/A

Software and algorithms		

GraphPad Prism 9	GraphPad	https://www.graphpad.com/
GSNAP V2021–12–17	Wu and Nacu^98^	http://research-pub.gene.com/gmap/
Cufflinks	Trapnell et al.^99^	https://github.com/santosjorge/cufflinks
R	The R Foundation	https://www.r-project.org/
PC-TraFF	Meckbach et al.^54^	http://pctraff.bioinf.med.uni-goettingen.de/
MSigDB V7.5.1	UC San Diego and Broad Institute	https://www.gsea-msigdb.org/gsea/msigdb/
DAVID V2022q1	Laboratory of Human Retrovirology and Immunoinformatics	https://david.ncifcrf.gov/
Ingenuity pathway analysis (IPA)	Qiagen	Cat#830018
MetaboAnalyst V5.0	OmicsForum	https://www.metaboanalyst.ca/
VistaVision	ISS, Inc.	https://iss.com/software/vistavision
SimFCS V4	Laboratory for Fluorescence Dynamics, University of California	https://www.lfd.uci.edu/globals/
Bowtie2	Langmead and Salzberg^100^	https://bowtie-bio.sourceforge.net/bowtie2/index.shtml
MACS2 V2.2.7.1	Zhang et al.^101^	https://github.com/macs3-project/MACS
FlowJo V10.7.1	BD Biosciences	https://www.flowjo.com/
ImageJ V1.53c	NIH	https://imagej.net/ij/index.html
SAS V9.4	SAS	https://www.sas.com/en_us/home.html

Other		

Phagocytosis 96-well plates	Greiner	Cat#655090
MACS MS columns	Miltenyi Biotec	Cat#130–042–201
35 mm high μ-dish	Ibidi	Cat#81156

## Highlights

- Maternal Western-style diet (mWSD) produces pro-inflammatory macrophages in juvenile non-human primates
- Metabolism/functional potential of mWSD-exposed fetal HSPCs mirrors juvenile HSPCs
- mWSD increases chromatin accessibility in juvenile HSPC and BMDM inflammatory genes
- Oleic acid increases in bone marrow of mWSD-exposed fetuses and juveniles

## References

[1] Brumbaugh DE, and Friedman JE (2014). Developmental origins of nonalcoholic fatty liver disease. Pediatr. Res 75, 140–147. 10.1038/pr.2013.193. 24192698 PMC4081536

[2] Godfrey KM, Reynolds RM, Prescott SL, Nyirenda M, Jaddoe VWV, Eriksson JG, and Broekman BFP (2017). Influence of maternal obesity on the long-term health of offspring. Lancet Diabetes Endocrinol 5, 53–64. 10.1016/s2213-8587(16)30107-3. 27743978 PMC5245733

[3] Harris RA, Alcott CE, Sullivan EL, Takahashi D, McCurdy CE, Comstock S, Baquero K, Blundell P, Frias AE, Kahr M, (2016). Genomic variants associated with resistance to high fat diet induced obesity in a primate model. Sci. Rep 6, 36123. 10.1038/srep36123. 27811965 PMC5095882

[4] Inzani I, and Ozanne SE (2022). Programming by maternal obesity: a pathway to poor cardiometabolic health in the offspring. Proc. Nutr. Soc 81, 227–242. 10.1017/s0029665122001914. 35974421 PMC7613838

[5] Kahr MK, Antony KM, DelBeccaro M, Hu M, Aagaard KM, and Suter MA (2016). Increasing maternal obesity is associated with alterations in both maternal and neonatal thyroid hormone levels. Clin. Endocrinol 84, 551–557. 10.1111/cen.12974. PMC4789139 26562744

[6] Mandala A, Janssen RC, Palle S, Short KR, and Friedman JE (2020). Pediatric non-alcoholic fatty liver disease: nutritional origins and potential molecular mechanisms. Nutrients 12, 3166. 10.3390/nu12103166. 33081177 PMC7602751

[7] Singer K, and Lumeng CN (2017). The initiation of metabolic inflammation in childhood obesity. J. Clin. Invest 127, 65–73. 10.1172/jci88882. 28045405 PMC5199687

[8] Suter MA, Sangi-Haghpeykar H, Showalter L, Shope C, Hu M, Brown K, Williams S, Harris RA, Grove KL, Lane RH, and Aagaard KM (2012). Maternal high-fat diet modulates the fetal thyroid axis and thyroid gene expression in a nonhuman primate model. Mol. Endocrinol 26, 2071–2080. 10.1210/me.2012-1214. 23015752 PMC3517714

[9] Suter MA, Takahashi D, Grove KL, and Aagaard KM (2013). Post-weaning exposure to a high-fat diet is associated with alterations to the hepatic histone code in Japanese macaques. Pediatr. Res 74, 252–258. 10.1038/pr.2013.106. 23788059 PMC3766448

[10] Wesolowski SR, Kasmi KCE, Jonscher KR, and Friedman JE (2017). Developmental origins of NAFLD: a womb with a clue. Nat. Rev. Gastroenterol. Hepatol 14, 81–96. 10.1038/nrgastro.2016.160. 27780972 PMC5725959

[11] Furman D, Campisi J, Verdin E, Carrera-Bastos P, Targ S, Franceschi C, Ferrucci L, Gilroy DW, Fasano A, Miller GW, (2019). Chronic inflammation in the etiology of disease across the life span. Nat. Med 25, 1822–1832. 10.1038/s41591-019-0675-0. 31806905 PMC7147972

[12] Renz H, Holt PG, Inouye M, Logan AC, Prescott SL, and Sly PD (2017). An exposome perspective: early-life events and immune development in a changing world. J. Allergy Clin. Immunol 140, 24–40. 10.1016/j.jaci.2017.05.015. 28673401

[13] Sureshchandra S, Mendoza N, Jankeel A, Wilson RM, Marshall NE, and Messaoudi I (2021). Phenotypic and epigenetic adaptations of cord blood CD4+ T cells to maternal obesity. Front. Immunol 12, 617592. 10.3389/fimmu.2021.617592. 33912153 PMC8071865

[14] Enninga EAL, Jang JS, Hur B, Johnson EL, Wick MJ, Sung J, and Chakraborty R (2021). Maternal obesity is associated with phenotypic alterations in fetal immune cells by single-cell mass cytometry. Am. J. Reprod. Immunol 85, e13358. 10.1111/aji.13358. 33064324 PMC8109213

[15] Christ A, Günther P, Lauterbach MAR, Duewell P, Biswas D, Pelka K, Scholz CJ, Oosting M, Haendler K, Baßler K, (2018). triggers NLRP3-dependent innate immune reprogramming. Cell 172, 162–175.e14. 10.1016/j.cell.2017.12.013. 29328911 PMC6324559

[16] de Laval B, Maurizio J, Kandalla PK, Brisou G, Simonnet L, Huber C, Gimenez G, Matcovitch-Natan O, Reinhardt S, David E, (2020). C/EBPβ-dependent epigenetic memory induces trained immunity in hematopoietic stem cells. Cell Stem Cell 26, 657–674.e8. 10.1016/j.stem.2020.01.017. 32169166

[17] Larsen SB, Cowley CJ, Sajjath SM, Barrows D, Yang Y, Carroll TS, and Fuchs E (2021). Establishment, maintenance, and recall of inflammatory memory. Cell Stem Cell 28, 1758–1774.e8. 10.1016/j.stem.2021.07.001. 34320411 PMC8500942

[18] Jentho E, Ruiz-Moreno C, Novakovic B, Kourtzelis I, Megchelenbrink WL, Martins R, Chavakis T, Soares MP, Kalafati L, Guerra J, (2021). Trained innate immunity, long-lasting epigenetic modulation, and skewed myelopoiesis by heme. Proc. Natl. Acad. Sci. USA 118, e2102698118. 10.1073/pnas.2102698118. 34663697 PMC8545490

[19] Netea MG, Domínguez-Andrés J, Barreiro LB, Chavakis T, Divangahi M, Fuchs E, Joosten LAB, van der Meer JWM, Mhlanga MM, Mulder WJM, (2020). Defining trained immunity and its role in health and disease. Nat. Rev. Immunol 20, 375–388. 10.1038/s41577-020-0285-6. 32132681 PMC7186935

[20] Kamimae-Lanning AN, Krasnow SM, Goloviznina NA, Zhu X, Roth-Carter QR, Levasseur PR, Jeng S, McWeeney SK, Kurre P, and Marks DL (2015). Maternal high-fat diet and obesity compromise fetal hematopoiesis. Mol. Metab 4, 25–38. 10.1016/j.molmet.2014.11.001. 25685687 PMC4314531

[21] Farley D, Tejero ME, Comuzzie AG, Higgins PB, Cox L, Werner SL, Jenkins SL, Li C, Choi J, Dick EJ Jr., (2009). Fetoplacental adaptations to maternal obesity in the baboon. Placenta 30, 752–760. 10.1016/j.placenta.2009.06.007. 19632719 PMC3011231

[22] Mouralidarane A, Soeda J, Visconti-Pugmire C, Samuelsson AM, Pombo J, Maragkoudaki X, Butt A, Saraswati R, Novelli M, Fusai G, (2013). Maternal obesity programs offspring nonalcoholic fatty liver disease by innate immune dysfunction in mice. Hepatology 58, 128–138. 10.1002/hep.26248. 23315950

[23] Odaka Y, Nakano M, Tanaka T, Kaburagi T, Yoshino H, Sato-Mito N, and Sato K (2010). The influence of a high-fat dietary environment in the fetal period on postnatal metabolic and immune function. Obesity 18, 1688–1694. 10.1038/oby.2009.513. 20111014

[24] Thorn SR, Baquero KC, Newsom SA, El Kasmi KC, Bergman BC, Shulman GI, Grove KL, and Friedman JE (2014). Early life exposure to maternal insulin resistance has persistent effects on hepatic NAFLD in juvenile nonhuman primates. Diabetes 63, 2702–2713. 10.2337/db14-0276. 24705404 PMC4113070

[25] López DA, Apostol AC, Lebish EJ, Valencia CH, Romero-Mulero MC, Pavlovich PV, Hernandez GE, Forsberg EC, Cabezas-Wallscheid N, and Beaudin AE (2022). Prenatal inflammation perturbs murine fetal hematopoietic development and causes persistent changes to postnatal immunity. Cell Rep 41, 111677. 10.1016/j.cel-rep.2022.111677. 36417858 PMC10184520

[26] Hata M, Andriessen EMMA, Hata M, Diaz-Marin R, Fournier F, Crespo-Garcia S, Blot G, Juneau R, Pilon F, Dejda A, (2023). Past history of obesity triggers persistent epigenetic changes in innate immunity and exacerbates neuroinflammation. Science 379, 45–62. 10.1126/science.abj8894. 36603072

[27] McCurdy CE, Bishop JM, Williams SM, Grayson BE, Smith MS, Friedman JE, and Grove KL (2009). Maternal high-fat diet triggers lipotoxicity in the fetal livers of nonhuman primates. J. Clin. Invest 119, 323–335. 10.1172/JCI32661. 19147984 PMC2631287

[28] Grant WF, Gillingham MB, Batra AK, Fewkes NM, Comstock SM, Takahashi D, Braun TP, Grove KL, Friedman JE, and Marks DL (2011). Maternal high fat diet is associated with decreased plasma n-3 fatty acids and fetal hepatic apoptosis in nonhuman primates. PLoS One 6, e17261. 10.1371/journal.pone.0017261. 21364873 PMC3045408

[29] Suter M, Bocock P, Showalter L, Hu M, Shope C, McKnight R, Grove K, Lane R, and Aagaard-Tillery K (2011). Epigenomics: maternal high-fat diet exposure in utero disrupts peripheral circadian gene expression in nonhuman primates. Faseb. J 25, 714–726. 10.1096/fj.10-172080. 21097519 PMC3228348

[30] Ma J, Prince AL, Bader D, Hu M, Ganu R, Baquero K, Blundell P, Alan Harris R, Frias AE, Grove KL, and Aagaard KM (2014). High-fat maternal diet during pregnancy persistently alters the offspring microbiome in a primate model. Nat. Commun 5, 3889. 10.1038/ncomms4889. 24846660 PMC4078997

[31] McCurdy CE, Schenk S, Hetrick B, Houck J, Drew BG, Kaye S, Lashbrook M, Bergman BC, Takahashi DL, Dean TA, (2016). Maternal obesity reduces oxidative capacity in fetal skeletal muscle of Japanese macaques. JCI Insight 1, e86612. 10.1172/jci.insight.86612. 27734025 PMC5053156

[32] Elsakr JM, Dunn JC, Tennant K, Zhao SK, Kroeten K, Pasek RC, Takahashi DL, Dean TA, Velez Edwards DR, McCurdy CE, (2019). Maternal Western-style diet affects offspring islet composition and function in a non-human primate model of maternal over-nutrition. Mol. Metab 25, 73–82. 10.1016/j.molmet.2019.03.010. 31036449 PMC6599455

[33] Wesolowski SR, Mulligan CM, Janssen RC, Baker PR, Bergman BC, D’Alessandro A, Nemkov T, Maclean KN, Jiang H, Dean TA, (2018). Switching obese mothers to a healthy diet improves fetal hypoxemia, hepatic metabolites, and lipotoxicity in non-human primates. Mol. Metab 18, 25–41. 10.1016/j.molmet.2018.09.008. 30337225 PMC6308036

[34] Campodonico-Burnett W, Hetrick B, Wesolowski SR, Schenk S, Takahashi DL, Dean TA, Sullivan EL, Kievit P, Gannon M, Aagaard K, (2020). Maternal obesity and Western-style diet impair fetal and juvenile offspring skeletal muscle insulin-stimulated glucose transport in nonhuman primates. Diabetes 69, 1389–1400. 10.2337/db19-1218. 32354857 PMC7306120

[35] Messaoudi I, Estep R, Robinson B, and Wong SW (2011). Nonhuman primate models of human immunology. Antioxid. Redox Signal 14, 261–273. 10.1089/ars.2010.3241. 20524846 PMC3014769

[36] Bishop CV, Takahashi D, Mishler E, Slayden OD, Roberts CT, Hennebold J, and True C (2021). Individual and combined effects of 5-year exposure to hyperandrogenemia and Western-style diet on metabolism and reproduction in female rhesus macaques. Hum. Reprod 36, 444–454. 10.1093/humrep/deaa321. 33313720 PMC7829549

[37] Kuo K, Roberts VHJ, Gaffney J, Takahashi DL, Morgan T, Lo JO, Stouffer RL, and Frias AE (2019). Maternal high-fat diet consumption and chronic hyperandrogenemia are associated with placental dysfunction in female rhesus macaques. Endocrinology 160, 1937–1949. 10.1210/en.2019-00149. 31180495 PMC6656425

[38] Gordon S (2003). Alternative activation of macrophages. Nat. Rev. Immunol 3, 23–35. 10.1038/nri978. 12511873

[39] Wynn TA, and Vannella KM (2016). Macrophages in tissue repair, regeneration, and fibrosis. Immunity 44, 450–462. 10.1016/j.immuni.2016.02.015. 26982353 PMC4794754

[40] McCowan J, Fercoq F, Kirkwood PM, T’Jonck W, Hegarty LM, Mawer CM, Cunningham R, Mirchandani AS, Hoy A, Humphries DC, (2021). The transcription factor EGR2 is indispensable for tissue-specific imprinting of alveolar macrophages in health and tissue repair. Sci. Immunol 6, eabj2132. 10.1126/sciimmunol.abj2132. 34797692 PMC7612216

[41] Viola A, Munari F, Sánchez-Rodríguez R, Scolaro T, and Castegna A (2019). The metabolic signature of macrophage responses. Front. Immunol 10, 1462. 10.3389/fimmu.2019.01462. 31333642 PMC6618143

[42] Stringari C, Nourse JL, Flanagan LA, and Gratton E (2012). Phasor fluorescence lifetime microscopy of free and protein-bound NADH reveals neural stem cell differentiation potential. PLoS One 7, e48014. 10.1371/journal.pone.0048014. 23144844 PMC3489895

[43] Chakraborty S, Nian FS, Tsai JW, Karmenyan A, and Chiou A (2016). Quantification of the metabolic state in cell-model of Parkinson’s disease by fluorescence lifetime imaging microscopy. Sci. Rep 6, 19145. 10.1038/srep19145. 26758390 PMC4725947

[44] Wallrabe H, Svindrych Z, Alam SR, Siller KH, Wang T, Kashatus D, Hu S, and Periasamy A (2018). Segmented cell analyses to measure redox states of autofluorescent NAD(P)H, FAD & Trp in cancer cells by FLIM. Sci. Rep 8, 79. 10.1038/s41598-017-18634-x. 29311591 PMC5758727

[45] Kumar R, Singh P, Kolloli A, Shi L, Bushkin Y, Tyagi S, and Subbian S (2019). Immunometabolism of phagocytes during mycobacterium tuberculosis infection. Front. Mol. Biosci 6, 105. 10.3389/fmolb.2019.00105. 31681793 PMC6803600

[46] Singer K, DelProposto J, Morris DL, Zamarron B, Mergian T, Maley N, Cho KW, Geletka L, Subbaiah P, Muir L, (2014). Diet-induced obesity promotes myelopoiesis in hematopoieticstem cells. Mol. Metab 3, 664–675. 10.1016/j.molmet.2014.06.005. 25161889 PMC4142398

[47] Chen L, and Ozato K (2021). Innate immune memory in hematopoietic stem/progenitor cells: myeloid-biased differentiation and the role of interferon. Front. Immunol 12, 621333. 10.3389/fimmu.2021.621333. 33854500 PMC8039377

[48] Yuan S, Liu Z, Xu Z, Liu J, and Zhang J (2020). High mobility group box 1 (HMGB1): a pivotal regulator of hematopoietic malignancies. J. Hematol. Oncol 13, 91. 10.1186/s13045-020-00920-3. 32660524 PMC7359022

[49] Laurenti E, Varnum-Finney B, Wilson A, Ferrero I, Blanco-Bose WE, Ehninger A, Knoepfler PS, Cheng PF, MacDonald HR, Eisenman RN, (2008). Hematopoietic stem cell function and survival depend on c-Myc and N-Myc activity. Cell Stem Cell 3, 611–624. 10.1016/j.stem.2008.09.005. 19041778 PMC2635113

[50] Park JH, Ku HJ, Lee JH, and Park JW (2018). Disruption of IDH2 attenuates lipopolysaccharide-induced inflammation and lung injury in an α-ketoglutarate-dependent manner. Biochem. Biophys. Res. Commun 503, 798–802. 10.1016/j.bbrc.2018.06.078. 29913148

[51] Seim GL, Britt EC, John SV, Yeo FJ, Johnson AR, Eisenstein RS, Pagliarini DJ, and Fan J (2019). Two-stage metabolic remodelling in macrophages in response to lipopolysaccharide and interferon-γ stimulation. Nat. Metab 1, 731–742. 10.1038/s42255-019-0083-2. 32259027 PMC7108803

[52] Blank U, and Karlsson S (2015). TGF-β signaling in the control of hematopoietic stem cells. Blood 125, 3542–3550. 10.1182/blood-2014-12-618090. 25833962

[53] Funnell APW, Norton LJ, Mak KS, Burdach J, Artuz CM, Twine NA, Wilkins MR, Power CA, Hung TT, Perdomo J, (2012). The CACCC-binding protein KLF3/BKLF represses a subset of KLF1/EKLF target genes and is required for proper erythroid maturation in vivo. Mol. Cell Biol 32, 3281–3292. 10.1128/mcb.00173-12. 22711990 PMC3434552

[54] Meckbach C, Tacke R, Hua X, Waack S, Wingender E, and Gültas M (2015). PC-TraFF: identification of potentially collaborating transcription factors using pointwise mutual information. BMC Bioinf 16, 400. 10.1186/s12859-015-0827-2. PMC4667426 26627005

[55] Orecchioni M, Ghosheh Y, Pramod AB, and Ley K (2019). Macrophage polarization: different gene signatures in M1(LPS+) vs. classically and M2(LPS-) vs. alternatively activated macrophages. Front. Immunol 10, 1084. 10.3389/fimmu.2019.01084. 31178859 PMC6543837

[56] Wilkinson AC, Morita M, Nakauchi H, and Yamazaki S (2018). Branched-chain amino acid depletion conditions bone marrow for hematopoietic stem cell transplantation avoiding amino acid imbalance-associated toxicity. Exp. Hematol 63, 12–16.e1. 10.1016/j.exphem.2018.04.004. 29705267 PMC6052250

[57] Pernes G, Flynn MC, Lancaster GI, and Murphy AJ (2019). Fat for fuel: lipid metabolism in haematopoiesis. Clin. Transl. Immunology 8, e1098. 10.1002/cti2.1098. 31890207 PMC6928762

[58] Hisha H, Yamada H, Sakurai MH, Kiyohara H, Li Y, Yu C, Takemoto N, Kawamura H, Yamaura K, Shinohara S, (1997). Isolation and identification of hematopoietic stem cell-stimulating substances from Kampo (Japanese herbal) medicine, Juzen-taiho-to. Blood 90, 1022–1030. 9242532

[59] Mistry JJ, Hellmich C, Moore JA, Jibril A, Macaulay I, Moreno-Gonzalez M, Di Palma F, Beraza N, Bowles KM, and Rushworth SA (2021). Free fatty-acid transport via CD36 drives β-oxidation-mediated hematopoietic stem cell response to infection. Nat. Commun 12, 7130. 10.1038/s41467-021-27460-9. 34880245 PMC8655073

[60] Hoggatt J, and Pelus LM (2010). Eicosanoid regulation of hematopoiesis and hematopoietic stem and progenitor trafficking. Leukemia 24, 1993–2002. 10.1038/leu.2010.216. 20882043 PMC3099594

[61] Maryanovich M, and Ito K (2022). CD36-mediated fatty acid oxidation in hematopoietic stem cells Is a novel mechanism of emergency hematopoiesis in response to infection. Immunometabolism 4, e220008. 10.20900/immunometab20220008. 35465142 PMC9029143

[62] Ito K, Carracedo A, Weiss D, Arai F, Ala U, Avigan DE, Schafer ZT, Evans RM, Suda T, Lee CH, and Pandolfi PP (2012). A PML–PPAR-d pathway for fatty acid oxidation regulates hematopoietic stem cell maintenance. Nat. Med 18, 1350–1358. 10.1038/nm.2882. 22902876 PMC3566224

[63] Robino JJ, Pamir N, Rosario S, Crawford LB, Burwitz BJ, Roberts CT Jr., Kurre P, and Varlamov O (2020). Spatial and biochemical interactions between bone marrow adipose tissue and hematopoietic stem and progenitor cells in rhesus macaques. Bone 133, 115248. 10.1016/j.bone.2020.115248. 31972314 PMC7085416

[64] Aguilar-Navarro AG, Meza-León B, Gratzinger D, Juárez-Aguilar FG, Chang Q, Ornatsky O, Tsui H, Esquivel-Gómez R, Hernán-dez-Ramírez A, Xie SZ, (2020). Human aging alters the spatial organization between CD34+ hematopoietic cells and adipocytes in bone marrow. Stem Cell Rep 15, 317–325. 10.1016/j.stemcr.2020.06.011. PMC7419665 32649902

[65] van der Heijden CDCC, Noz MP, Joosten LAB, Netea MG, Riksen NP, and Keating ST (2018). Epigenetics and trained immunity. Antioxid. Redox Signal 29, 1023–1040. 10.1089/ars.2017.7310. 28978221 PMC6121175

[66] Fanucchi S, and Mhlanga MM (2019). Lnc-ing trained immunity to chromatin architecture. Front. Cell Dev. Biol 7, 2. 10.3389/fcell.2019.00002. 30733945 PMC6353842

[67] Fanucchi S, Domínguez-Andrés J, Joosten LAB, Netea MG, and Mhlanga MM (2021). The intersection of epigenetics and metabolism in trained immunity. Immunity 54, 32–43. 10.1016/j.im-muni.2020.10.011. 33220235

[68] Sureshchandra S, Chan CN, Robino JJ, Parmelee LK, Nash MJ, Wesolowski SR, Pietras EM, Friedman JE, Takahashi D, Shen W, (2022). Maternal Western-style diet remodels the transcriptional landscape of fetal hematopoietic stem and progenitor cells in rhesus macaques. Stem Cell Rep 17, 2595–2609. 10.1016/j.stemcr.2022.10.003. PMC9768582 36332628

[69] Mitroulis I, Ruppova K, Wang B, Chen LS, Grzybek M, Grinenko T, Eugster A, Troullinaki M, Palladini A, Kourtzelis I, (2018). Modulation of myelopoiesis progenitors is an integral component of trained immunity. Cell 172, 147–161.e12. 10.1016/j.cell.2017.11.034. 29328910 PMC5766828

[70] Nash MJ, Dobrinskikh E, Newsom SA, Messaoudi I, Janssen RC, Aagaard KM, McCurdy CE, Gannon M, Kievit P, Friedman JE, and Wesolowski SR (2021). Maternal Western diet exposure increases periportal fibrosis beginning in utero in nonhuman primate offspring. JCI Insight 6, e154093. 10.1172/jci.insight.154093. 34935645 PMC8783685

[71] Soares-da-Silva F, Peixoto M, Cumano A, and Pinto-do-Ó P (2020). Crosstalk between the hepatic and hematopoietic systems during embryonic development. Front. Cell Dev. Biol 8, 612. 10.3389/fcell.2020.00612. 32793589 PMC7387668

[72] Smith JNP, and Calvi LM (2013). Concise review: current concepts in bone marrow microenvironmental regulation of hematopoietic stem and progenitor cells. Stem Cell 31, 1044–1050. 10.1002/stem.1370. PMC3664122 23509002

[73] Wielockx B, Grinenko T, Mirtschink P, and Chavakis T (2019). Hypoxia pathway proteins in normal and malignant hematopoiesis. Cells 8, 155. 10.3390/cells8020155. 30781787 PMC6406588

[74] Wang T, Liu H, Lian G, Zhang SY, Wang X, and Jiang C (2017). HIF1α-induced glycolysis metabolism is essential to the activation of inflammatory macrophages. Mediators Inflamm 2017, 9029327. 10.1155/2017/9029327. 29386753 PMC5745720

[75] Naveiras O, Nardi V, Wenzel PL, Hauschka PV, Fahey F, and Daley GQ (2009). Bone-marrow adipocytes as negative regulators of the haematopoietic microenvironment. Nature 460, 259–263. 10.1038/nature08099. 19516257 PMC2831539

[76] Wang H, Leng Y, and Gong Y (2018). Bone marrow fat and hematopoiesis. Front. Endocrinol 9, 694. 10.3389/fendo.2018.00694. PMC6280186 30546345

[77] Gensollen T, Iyer SS, Kasper DL, and Blumberg RS (2016). How colonization by microbiota in early life shapes the immune system. Science 352, 539–544. 10.1126/science.aad9378. 27126036 PMC5050524

[78] Chavakis T, Mitroulis I, and Hajishengallis G (2019). Hematopoieticpro-genitor cells as integrative hubs for adaptation to and fine-tuning of inflammation. Nat. Immunol 20, 802–811. 10.1038/s41590-019-0402-5. 31213716 PMC6709414

[79] Macpherson AJ, de Agüero MG, and Ganal-Vonarburg SC (2017). How nutrition and the maternal microbiota shape the neonatal immune system. Nat. Rev. Immunol 17, 508–517. 10.1038/nri.2017.58. 28604736

[80] Aagaard K, Ma J, Antony KM, Ganu R, Petrosino J, and Versalovic J (2014). The placenta harbors a unique microbiome. Sci. Transl. Med 6, 237ra65. 10.1126/scitranslmed.3008599. PMC4929217 24848255

[81] Rodríguez JM (2014). The origin of human milk bacteria: is there a bacterial entero-mammary pathway during late pregnancy and lactation? Adv. Nutr 5, 779–784. 10.3945/an.114.007229. 25398740 PMC4224214

[82] Sindi AS, Stinson LF, Lean SS, Chooi YH, Leghi GE, Netting MJ, Wlodek ME, Muhlhausler BS, Geddes DT, and Payne MS (2022). Effect of a reduced fat and sugar maternal dietary intervention during lactation on the infant gut microbiome. Front. Microbiol 13, 900702. 10.3389/fmicb.2022.900702. 36060782 PMC9428759

[83] Gorski JN, Dunn-Meynell AA, Hartman TG, and Levin BE (2006). Postnatal environment overrides genetic and prenatal factors influencing offspring obesity and insulin resistance. Am. J. Physiol. Regul. Integr. Comp. Physiol 291, R768–R778. 10.1152/aj-pregu.00138.2006. 16614055

[84] Gomes RM, Bueno FG, Schamber CR, de Mello JCP, de Oliveira JC, Francisco FA, Moreira VM, Junior MDF, Pedrino GR, de Freitas Mathias PC, (2018). Maternal diet-induced obesity during suckling period programs offspring obese phenotype and hypothalamic leptin/insulin resistance. J. Nutr. Biochem 61, 24–32. 10.1016/j.jnutbio.2018.07.006. 30179726

[85] Pomar CA, Castillo P, Palou M, Palou A, and Picó C (2022). Implementation of a healthy diet to lactating rats attenuates the early detrimental programming effects in the offspring born to obese dams. Putative relationship with milk hormone levels. J. Nutr. Biochem 107, 109043. 10.1016/j.jnutbio.2022.109043. 35569798

[86] Castillo P, Pomar CA, Palou A, Palou M, and Picó C (2023). Influence of maternal metabolic status and diet during the perinatal period on the metabolic programming by leptin ingested during the suckling period in rats. Nutrients 15, 570. 10.3390/nu15030570. 36771278 PMC9921535

[87] Roberts VHJ, Pound LD, Thorn SR, Gillingham MB, Thornburg KL, Friedman JE, Frias AE, and Grove KL (2014). Beneficial and cautionary outcomes of resveratrol supplementation in pregnant nonhuman primates. Faseb. J 28, 2466–2477. 10.1096/fj.13-245472. 24563374 PMC4021444

[88] Küpers LK, Fernández-Barrés S, Mancano G, Johnson L, Ott R, Vioque J, Colombo M, Landgraf K, Tobi EW, Körner A, (2022). Maternal dietary glycemic index and glycemic load in pregnancy and offspring cord blood DNA methylation. Diabetes Care 45, 1822–1832. 10.2337/dc21-2662. 35708509 PMC9346994

[89] Monasso GS, Voortman T, and Felix JF (2022). Maternal plasma fatty acid patterns in mid-pregnancy and offspring epigenetic gestational age at birth. Epigenetics 17, 1562–1572. 10.1080/15592294.2022.2076051. 35581922 PMC9586633

[90] Robinson SL, Mumford SL, Guan W, Zeng X, Kim K, Radoc JG, Trinh MH, Flannagan K, Schisterman EF, and Yeung E (2020). Maternal fatty acid concentrations and newborn DNA methylation. Am. J. Clin. Nutr 111, 613–621. 10.1093/ajcn/nqz311. 31858113 PMC7049533

[91] Aagaard-Tillery KM, Grove K, Bishop J, Ke X, Fu Q, McKnight R, and Lane RH (2008). Developmental origins of disease and determinants of chromatin structure: maternal diet modifies the primate fetal epigenome. J. Mol. Endocrinol 41, 91–102. 10.1677/jme-08-0025. 18515302 PMC2959100

[92] Suter MA, Chen A, Burdine MS, Choudhury M, Harris RA, Lane RH, Friedman JE, Grove KL, Tackett AJ, and Aagaard KM (2012). A maternal high-fat diet modulates fetal SIRT1 histone and protein deacetylase activity in nonhuman primates. Faseb. J 26, 5106–5114. 10.1096/fj.12-212878. 22982377 PMC3509051

[93] Nash MJ, Dobrinskikh E, Janssen RC, Lovell MA, Schady DA, Levek C, Jones KL, D’Alessandro A, Kievit P, Aagaard KM, (2023). Maternal Western diet is associated with distinct preclinical pediatric NAFLD phenotypes in juvenile nonhuman primate offspring. Hepatol. Commun 7, e0014. 10.1097/HC9.0000000000000014. 36691970 PMC9851700

[94] Schuster S, Cabrera D, Arrese M, and Feldstein AE (2018). Triggering and resolution of inflammation in NASH. Nat. Rev. Gastroenterol. Hepatol 15, 349–364. 10.1038/s41575-018-0009-6. 29740166

[95] Carter JK, and Friedman SL (2022). Hepatic stellate cell-immune interactions in NASH. Front. Endocrinol 13, 867940. 10.3389/fendo.2022.867940. PMC9218059 35757404

[96] Liu Y, Xu R, Gu H, Zhang E, Qu J, Cao W, Huang X, Yan H, He J, and Cai Z (2021). Metabolic reprogramming in macrophage responses. Biomark. Res 9, 1. 10.1186/s40364-020-00251-y. 33407885 PMC7786975

[97] Bodden C, Hannan AJ, and Reichelt AC (2020). Diet-induced modification of the sperm epigenome programs metabolism and behavior. Trends Endocrinol. Metab 31, 131–149. 10.1016/j.tem.2019.10.005. 31744784

[98] Wu TD, and Nacu S (2010). Fast and SNP-tolerant detection of complex variants and splicing in short reads. Bioinformatics 26, 873–881. 10.1093/bioinformatics/btq057. 20147302 PMC2844994

[99] Trapnell C, Williams BA, Pertea G, Mortazavi A, Kwan G, van Baren MJ, Salzberg SL, Wold BJ, and Pachter L (2010). Transcript assembly and quantification by RNA-Seq reveals unannotated transcripts and isoform switching during cell differentiation. Nat. Biotechnol 28, 511–515. 10.1038/nbt.1621. 20436464 PMC3146043

[100] Langmead B, and Salzberg SL (2012). Fast gapped-read alignment with Bowtie 2. Nat. Methods 9, 357–359. 10.1038/nmeth.1923. 22388286 PMC3322381

[101] Zhang Y, Liu T, Meyer CA, Eeckhoute J, Johnson DS, Bernstein BE, Nusbaum C, Myers RM, Brown M, Li W, and Liu XS (2008). Model-based analysis of ChIP-seq (MACS). Genome Biol 9, R137. 10.1186/gb-2008-9-9-r137. 18798982 PMC2592715

[102] True CA, Takahashi DL, Burns SE, Mishler EC, Bond KR, Wilcox MC, Calhoun AR, Bader LA, Dean TA, Ryan ND, (2017). Chronic combined hyperandrogenemia and western-style diet in young female rhesus macaques causes greater metabolic impairments compared to either treatment alone. Hum. Reprod 32, 1880–1891. 10.1093/humrep/dex246. 28854721 PMC5850848

[103] Varlamov O, Bishop CV, Handu M, Takahashi D, Srinivasan S, White A, and Roberts CT Jr. (2017). Combined androgen excess and Western-style diet accelerates adipose tissue dysfunction in young adult, female nonhuman primates. Hum. Reprod 32, 1892–1902. 10.1093/humrep/dex244. 28854720 PMC6074799

[104] Elsakr JM, Zhao SK, Ricciardi V, Dean TA, Takahashi DL, Sullivan E, Wesolowski SR, McCurdy CE, Kievit P, Friedman JE, (2021). -style diet consumption impairs maternal insulin sensitivity and glucose metabolism during pregnancy in a Japanese macaque model. Sci. Rep 11, 12977. 10.1038/s41598-021-92464-w. 34155315 PMC8217225

[105] Skehan P, Storeng R, Scudiero D, Monks A, McMahon J, Vistica D, Warren JT, Bokesch H, Kenney S, and Boyd MR (1990). New colorimetric cytotoxicity assay for anticancer-drug screening. J. Natl. Cancer Inst 82, 1107–1112. 10.1093/jnci/82.13.1107. 2359136

[106] Vichai V, and Kirtikara K (2006). Sulforhodamine B colorimetric assay for cytotoxicity screening. Nat. Protoc 1, 1112–1116. 10.1038/nprot.2006.179. 17406391

[107] Baird NL, Bowlin JL, Cohrs RJ, Gilden D, and Jones KL (2014). Comparison of varicella-zoster virus RNA sequences in human neurons and fibroblasts. J. Virol 88, 5877–5880. 10.1128/jvi.00476-14. 24600007 PMC4019124

[108] Subramanian A, Tamayo P, Mootha VK, Mukherjee S, Ebert BL, Gillette MA, Paulovich A, Pomeroy SL, Golub TR, Lander ES, and Mesirov JP (2005). Gene set enrichment analysis: a knowledge-based approach for interpreting genome-wide expression profiles. Proc. Natl. Acad. Sci. USA 102, 15545–15550. 10.1073/pnas.0506580102. 16199517 PMC1239896

[109] Huang DW, Sherman BT, and Lempicki RA (2009). Systematic and integrative analysis of large gene lists using DAVID bioinformatics resources. Nat. Protoc 4, 44–57. 10.1038/nprot.2008.211. 19131956

[110] Radtke S, Adair JE, Giese MA, Chan YY, Norgaard ZK, Enstrom M, Haworth KG, Schefter LE, and Kiem HP (2017). A distinct hematopoietic stem cell population for rapid multilineage engraftment in nonhuman primates. Sci. Transl. Med 9, eaan1145. 10.1126/scitranslmed.aan1145. PMC6467214 29093179

[111] Marwan AI, Shabeka U, Reisz JA, Zheng C, Serkova NJ, and Dobrinskikh E (2019). Unique heterogeneous topological pattern of the metabolic landscape in rabbit fetal lungs following tracheal occlusion. Fetal Diagn. Ther 45, 145–154. 10.1159/000487752. 29669344 PMC6314905

[112] Dobrinskikh E, Al-Juboori SI, Shabeka U, Reisz JA, Zheng C, and Marwan AI (2019). Heterogeneous pulmonary response after tracheal occlusion: clues to fetal lung growth. J. Surg. Res 239, 242–252. 10.1016/j.jss.2019.02.015. 30856517

[113] Smith JP, Corces MR, Xu J, Reuter VP, Chang HY, and Sheffield NC (2021). PEPATAC: an optimized pipeline for ATAC-seq data analysis with serial alignments. NAR Genom. Bioinform 3, lqab101. 10.1093/nargab/lqab101. 34859208 PMC8632735

[114] Zarini S, Gijón MA, Folco G, and Murphy RC (2006). Effect of arachidonic acid reacylation on leukotriene biosynthesis in human neutrophils stimulated with granulocyte-macrophage colony-stimulating factor and formyl-methionyl-leucyl-phenylalanine. J. Biol. Chem 281, 10134–10142. 10.1074/jbc.M510783200. 16495221

