# Supplementary material for: Maternal diet alters long-term innate immune cell memory in fetal and juvenile hematopoietic stem and progenitor cells in nonhuman primate offspring
Source: Cell Rep. Author manuscript; Available in PMC 2023 Oct 23. (PMC10570400; doi:10.1016/j.celrep.2023.112393)
Supplement: 1 [file NIHMS1897947-supplement-1.pdf]

Cell Reports, Volume *42*

Supplemental information

Maternal diet alters long-term innate immune cell memory in fetal and juvenile hematopoietic stem and progenitor cells in nonhuman primate offspring

Michael J. Nash, Evgenia Dobrinskikh, Taylor K. Soderborg, Rachel C. Janssen, Diana L. Takahashi, Tyler A. Dean, Oleg Varlamov, Jon D. Hennebold, Maureen Gannon, Kjersti M. Aagaard, Carrie E. McCurdy, Paul Kievit, Bryan C. Bergman, Kenneth L. Jones, Eric M. Pietras, Stephanie R. Wesolowski, and Jacob E. Friedman

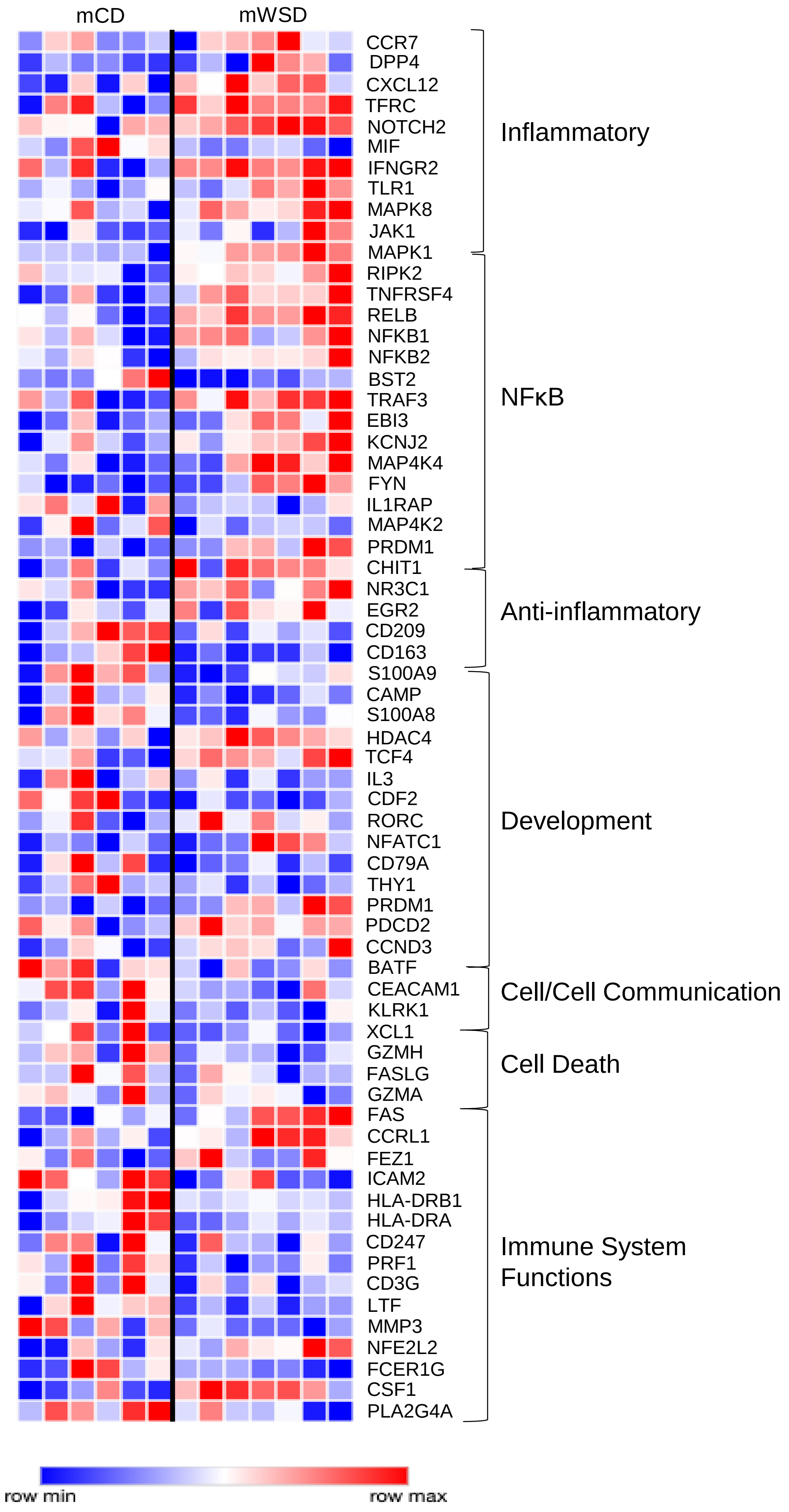

Figure S1. Heatmap showing differentially expressed genes (DEGs) ($p < 0.05$) in juvenile unstimulated BMDMs measured with NanoString gene expression analysis. DEGs are grouped by function. n = 6 mCD, n = 7 mWSD.

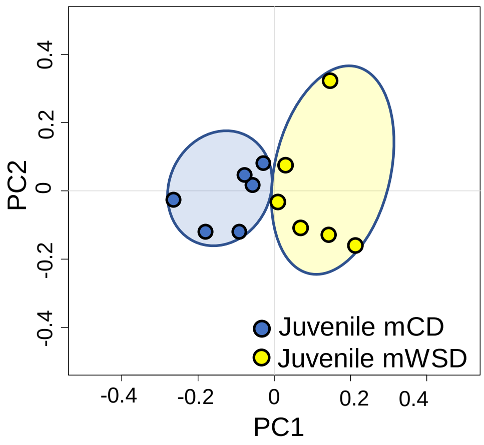

Figure S2. Principal component analysis of gene expression measured by bulk RNA-seq in mCD- vs. mWSD-exposed juvenile HSPCs. n = 6 mCD and n = 6 mWSD.

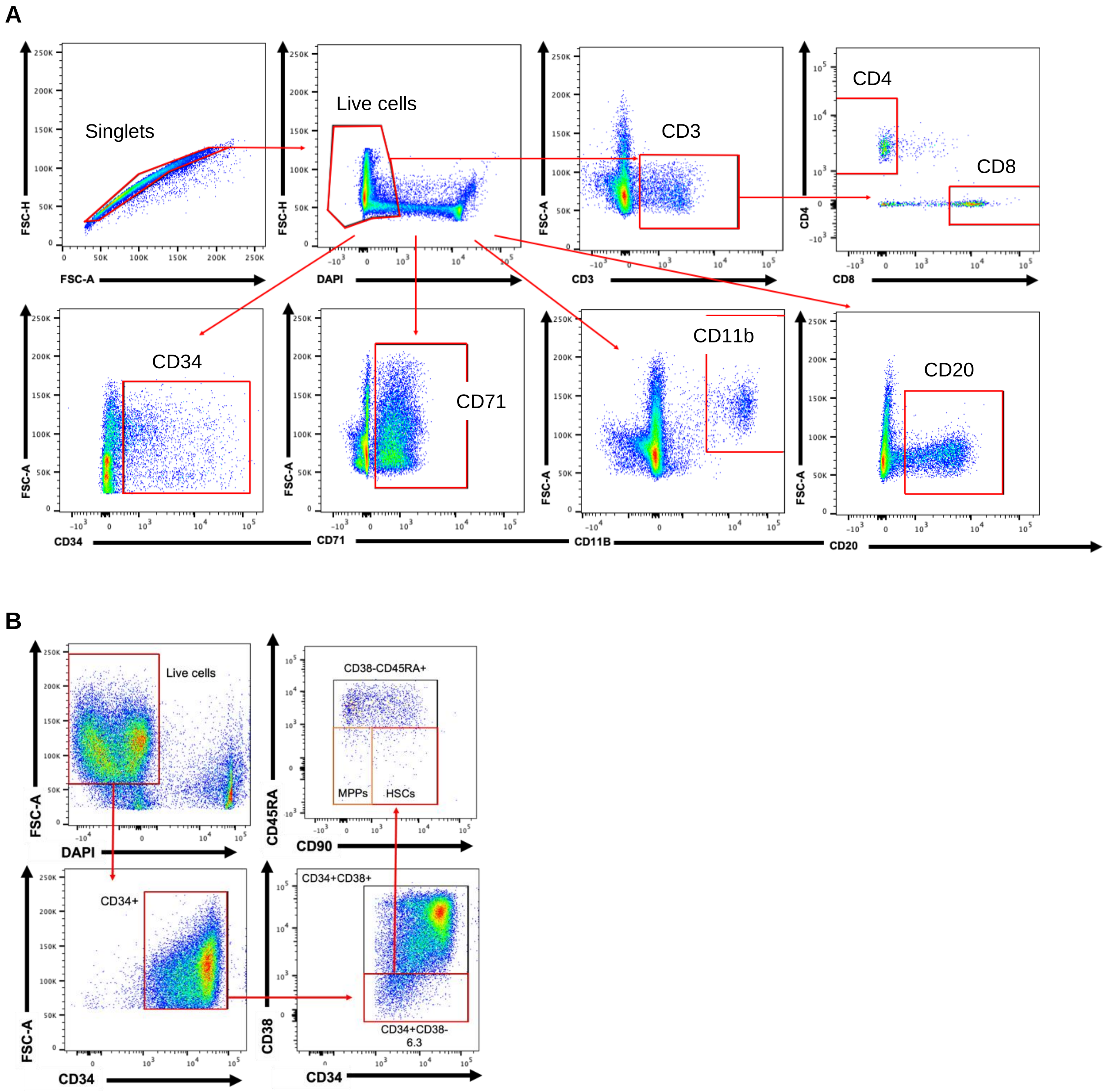

Figure S3. Gating strategies for flow cytometric analysis of bone marrow MNCs (**A**) and HSPCs (**B**). The same gating strategies were used for fetal and juvenile samples.

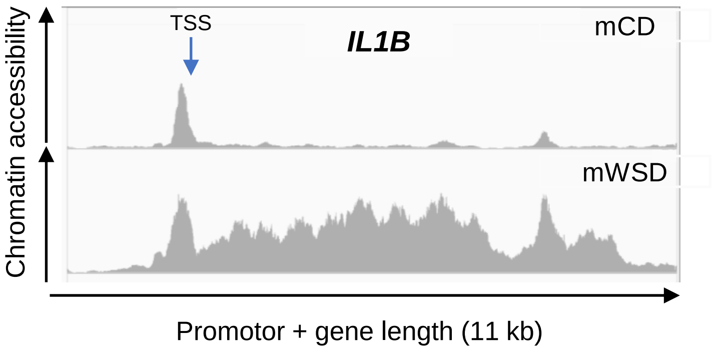

Figure S4. ATAC-seq chromatin reads on *IL1B* in mCD- and mWSD-exposed juvenile BMDMs. Chromatin read map of *IL1B*. Y-axis indicates ATAC-seq signal intensity which is comprised of reads, represented as peaks. The entire gene body for *IL1B* is shown. Average reads for n = 5 juvenile mCD and n = 5 juvenile mWSD are shown.

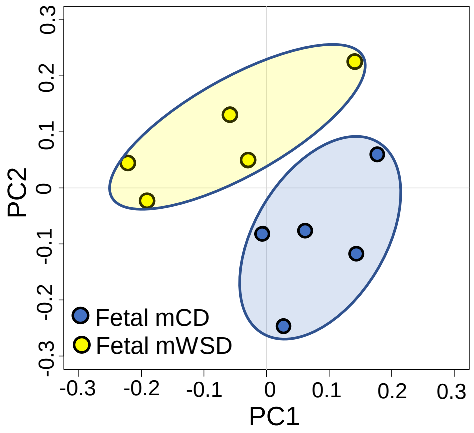

Figure S5. Principal component analysis of gene expression measured by bulk RNA-seq in mCD- vs. mWSD-exposed fetal bone marrow HSPCs. n = 5 mCD and n = 5 mWSD.

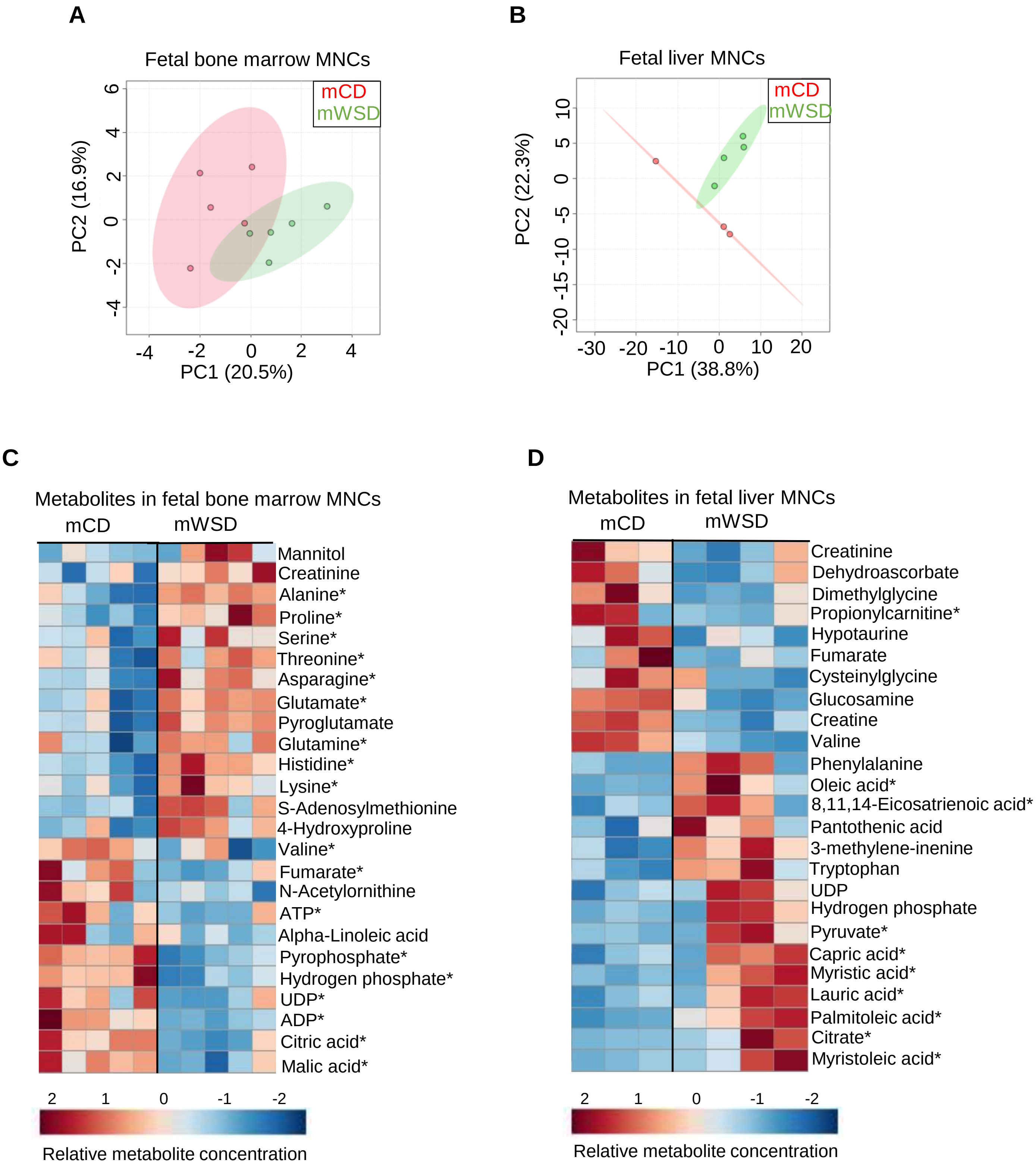

Figure S6. Principal component analysis of gene expression measured by bulk RNA-seq in mCD- vs. mWSD-exposed fetal bone marrow MNCs (**A**) and liver MNCs (**B**). Heatmaps of relative concentration of top 25 VIP scoring metabolites in fetal bone marrow MNCs (**C**) and liver MNCs (**D**). *p < 0.05 via Student's t test between fetal mCD and fetal mWSD. n = 5 mCD and n = 5 mWSD bone marrow MNCs; n = 3 mCD and n = 4 mWSD liver MNCs.

Table S1. Phenotype of rhesus macaque dams and fetuses.

	mCD	mWSD	p value
**Maternal**[a]			
Age, years	8.5 ± 0.1	8.5 ± 0.1	0.83
Body weight, kg	9.0 ± 0.4	10.1 ± 0.8	0.32
Body fat, %	36.5 ± 3.6	46.7 ± 2.8	**<0.05**
Glucose, mg/dL	54.2 ± 3.1	67.6 ± 9.3	0.23
Insulin, µU/mL	158 ± 76	394 ± 169	0.25
Insulin AUC	55906 ± 18418	62304 ± 10571	0.76
Glucose AUC	7964 ± 401	9280 ± 1071	0.30
**Fetal**[b]			
n	9	5	
Sex, female/male	7/2	3/2	
Body weight, g	349 ± 11	352 ± 31	0.93
Crown-rump length, cm	18.5 ± 0.3	19.2 ± 0.6	0.38
Liver weight, g	11.9 ± 1.6	12.1 ± 1.3	0.91
RWAT weight, mg	34.4 ± 4.7	52.5 ± 20.5	0.44
Glucose, mg/dL	33.1 ± 1.9	39.0 ± 5.1	0.33

[a]Age, body weight, and body fat were measured within six months prior to pregnancy. Intravenous glucose tolerance test was performed during fasting at 0.7 gestation: baseline plasma glucose and insulin were measured and the area under the curve (AUC) for glucose and insulin was calculated.
[b]Fetal values were obtained at necropsy (0.7 gestation).
Data are presented as mean ± SEM. p values from unpaired Student's t test are shown (significant p value in bold).
RWAT, retroperitoneal white adipose tissue.

Table S2. Phenotype of Japanese macaque dams and juvenile offspring.

	mCD	mWSD	p value
**Maternal**[a]			
Age, years	10.6 ± 0.9	8.9 ± 0.5	0.11
Body weight, kg	9.8 ± 0.4	11.5 ± 0.4	**<0.005**
Body fat, %	25.0 ± 1.6	33.3 ± 1.7	**<0.002**
Glucose, mg/dL	42.9 ± 2.6	40.7 ± 1.8	0.51
Insulin, µU/mL	38.8 ± 11.2	35.4 ± 7.2	0.80
Insulin AUC	7692 ± 980	12303 ± 2355	0.07
Glucose AUC	6245 ± 382	6343 ± 403	0.86
**Juvenile offspring**[b]			
n	19	15	
Sex, female/male	12/7	4/11	
Body weight, kg	6.2 ± 0.1	7.1 ± 0.3	**<0.05**
Fat mass, g	1005 ± 33	1133 ± 65	0.07
Body fat, %	15.8 ± 0.2	15.4 ± 0.3	0.35
Lean mass, g	5037 ± 147	5838 ± 233	**<0.005**
Glucose, mg/dL	58 ± 1.6	55 ± 2.0	0.22
Insulin, µU/mL	4.5 ± 0.7	7.1 ± 2.1	0.20
Glucose AUC	10031 ± 214	9552 ± 437	0.29
Insulin AUC	1697 ± 145	2037 ± 246	0.21
Liver weight, g[c]	144 ± 6	136 ± 3	0.45
RWAT weight, g[c]	0.51 ± 0.08	1.32 ± 0.35	**<0.005**

[a]Age, body weight, and body fat were measured within six months prior to pregnancy. Intravenous glucose tolerance test was performed during fasting at 0.7 gestation: baseline plasma glucose and insulin were measured and the area under the curve (AUC) for glucose and insulin was calculated.
[b]Juvenile values (excluding tissue weights) were obtained two months prior to necropsy.
[c]Obtained at necropsy.
Data are presented as mean ± SEM. Unpaired Student's t test p values are shown (significant p value in bold).
RWAT, retroperitoneal white adipose tissue.

Table S3. Canonical pathways predicted from DEGs regulated by mWSD in juvenile HSPCs.

Canonical pathway name[a]	-Log(p value)	Z-score[b]	Predicted activation state[c]	# DEGs in pathway[d]
EIF2 Signaling	32.2	4.46	Up-regulated	73
mTOR Signaling	15.6	3.00	Up-regulated	50
Regulation of eIF4 and p70S6K Signaling	14.5	1.63	Up-regulated	44
Unfolded protein response	13.0	3.15	Up-regulated	29
Hypoxia Signaling in the Cardiovascular System	10.1	0.45	Up-regulated	23
Coronavirus Pathogenesis Pathway	10.0	-2.53	Down-regulated	40
Protein Ubiquitination Pathway	9.91	N/A		48
Huntington's Disease Signaling	9.58	1.60	Up-regulated	48
Spliceosomal Cycle	7.58	4.00	Up-regulated	16
Sirtuin Signaling Pathway	7.16	0.78	Up-regulated	44
NRF2-mediated Oxidative Stress Response	6.51	4.24	Up-regulated	37
Mitochondrial Dysfunction	6.50	N/A		30
Insulin Secretion Signaling Pathway	5.95	5.92	Up-regulated	39
Inflammasome pathway	5.86	3.00	Up-regulated	9
Polyamine Regulation in Colon Cancer	5.68	1.29	Up-regulated	15
Ferroptosis Signaling Pathway	5.62	2.04	Up-regulated	24

[a]Shown in rank order by p value.
[b]Negative Z-scores correspond to predicted decrease in canonical pathway activity; positive Z-scores correspond to predicted increase in canonical pathway activity. N/A indicates that IPA did not attempt to assign a Z-score to that pathway. Pathways with Z-scores of N/A therefore do not have predicted activation states listed.
[c]Predicted activation state is calculated by IPA software and indicates whether the canonical pathway activity is increased or decreased.
[d]The number of differentially expressed genes (DEGs) present in IPA's database, corresponding to each canonical pathway.
n = 5 juvenile mCD, n = 6 juvenile mWSD.

Table S4. Upstream regulators predicted from DEGs regulated by mWSD in juvenile HSPCs.

Upstream regulator name[a]	p value/-Log(p value)	Z-score[b]	Activation state[c]	# DEGs in pathway[d]
ST1926	32.2	-7.60	Down-regulated	68
CD 437	15.6	-7.51	Down-regulated	76
MYC	14.5	8.44	Up-regulated	209
5-fluorouracil	13.0	-4.46	Down-regulated	90
LARP1	10.1	-6.56	Down-regulated	44
HNF4A	10.0	1.16	Up-regulated	282
TP53	9.91	0.29	Up-regulated	261
torin1	9.58	-4.44	Down-regulated	62
sirolimus	7.58	-7.34	Down-regulated	113
TCR	7.16	4.67	Up-regulated	80
MYCN	6.51	4.72	Up-regulated	74
CD3	6.50	8.05	Up-regulated	109

[a]Shown in rank order by p value.
[b]Negative Z-scores correspond to predicted decrease in upstream regulator pathway activity; positive Z-scores correspond to predicted increase in upstream regulator pathway activity.
[c]Predicted activation state is calculated by IPA software and indicates whether the upstream regulator pathway activity is decreased or increased.
[d]The number of differentially expressed genes (DEGs) present in IPA's database, corresponding to each upstream regulator pathway.
n = 5 juvenile mCD, n = 6 juvenile mWSD.

Table S5. Juvenile offspring complete blood counts.

CBC variable	mCD	mWSD	p value
WBC, X 10^9/L	6.8 ± 0.8	5.4 ± 0.3	0.25
Neutrophils, %	59 ± 3.0	62 ± 2.0	0.50
Lymphocytes, %	35 ± 3.0	31 ± 2.0	0.48
Monocytes, %	4.1 ± 0.4	4.1 ± 0.5	0.95
Eosinophils, %	1.4 ± 0.1	1.6 ± 0.1	0.43
Basophils, %	0.33 ± 0.0	0.25 ± 0.0	0.06
Hematocrit, %	38.8 ± 0.5	39.3 ± 0.8	0.59
Hemoglobin, mmol/L	12.6 ± 0.1	12.9 ± 0.2	0.52
Red blood cells, X 10^{12}/L	4.7 ± 0.0	4.7 ± 0.1	0.97
MCV, fL	82 ± 0.7	83 ± 0.5	0.26
MCH, fmol/cell	26.8 ± 0.1	27.3 ± 0.1	0.11
MCHC, mmol/L	32.7 ± 0.1	32.8 ± 0.1	0.61
Mean platelet volume, fL	9.4 ± 0.1	9.4 ± 0.2	0.96
Platelets, X 10^9/L	345 ± 12	363 ± 14	0.38

CBC, complete blood count; WBC; white blood cells, MCV; mean corpuscular volume, MCH; mean corpuscular hemoglobin. MCHC; mean corpuscular hemoglobin concentration. % indicates percentage of WBC count.
Data are presented as mean ± SEM. Unpaired Student’s t test p values are shown. n = 19 juvenile mCD, n = 15 juvenile mWSD.

Table S6. Canonical pathways predicted from DORs in juvenile HSPCs and BMDMs.

Canonical pathway name[a]	-Log(p value)	Z-score[b]	Predicted activation state[c]	# DORs in pathway[d]
**HSPCs**				
Protein Ubiquitination Pathway	5.03	N/A		16
EIF2 Signaling	4.18	0		13
Regulation of eIF4 and p70S6K Signaling	3.84	N/A		11
Cardiolipin Biosynthesis II	3.60	N/A		2
PPARα/RXRα Activation	3.52	-1.41	Down-regulated	11
Estrogen Receptor Signaling	3.49	2.14	Up-regulated	17
NRF2-mediated Oxidative Stress Response	3.36	0.45	Up-regulated	12
Insulin Secretion Signaling Pathway	3.34	0		13
Huntington's Disease Signaling	3.19	N/A		13
Thiosulfate Disproportionation III (Rhodanese)	3.13	N/A		2
Phosphatidylglycerol Biosynthesis II (Non-plastidic)	3.08	0		4
**BMDMs**				
NRF2-mediated Oxidative Stress Response	7.09	2.5	Up-regulated	23
Role of Tissue Factor in Cancer	6.41	N/A		15
Ferroptosis Signaling Pathway	6.41	-0.5	Down-regulated	16
CLEAR Signaling Pathway	6.22	-1.63	Down-regulated	24
IL-8 Signaling	6.12	3	Up-regulated	20
Molecular Mechanisms of Cancer	5.98	N/A		31
TNFR2 Signaling	5.89	1.63	Up-regulated	8
Hepatic Fibrosis Signaling Pathway	5.56	1.96	Up-regulated	29
Hepatic Fibrosis / Hepatic Stellate Cell Activation	4.81	N/A		17
Differential Regulation of Cytokine Production in Intestinal Epithelial Cells by IL-17A and IL-17F	4.66	1.63	Up-regulated	6
PPAR Signaling	4.61	-2.31	Down-regulated	12
Glucocorticoid Receptor Signaling	4.50	N/A		33
TREM1 Signaling	4.49	2.53	Up-regulated	10
IL-6 Signaling	4.48	3.05	Up-regulated	13
IL-17 Signaling	4.44	2.5	Up-regulated	16
Neuroinflammation Signaling Pathway	4.43	2.56	Up-regulated	22
PPARα/RXRα Activation	4.22	-1.39	Down-regulated	16
CD40 Signaling	4.21	1	Up-regulated	9

Relaxin Signaling	4.21	1.26	Up-regulated	14
Role of Macrophages, Fibroblasts and Endothelial Cells in Rheumatoid Arthritis	4.21	N/A		22
Huntington's Disease Signaling	4.19	0.90	Up-regulated	20
Cholecystokinin/Gastrin-mediated Signaling	4.15	1.51	Up-regulated	12
p38 MAPK Signaling	4.12	1.15	Up-regulated	12
Differential Regulation of Cytokine Production in Macrophages and T Helper Cells by IL-17A and IL-17F	4.10	1.34	Up-regulated	5
Apoptosis Signaling	4.04	0.30	Up-regulated	11
Sirtuin Signaling Pathway	4.00	0		20

[a]Shown in rank order by *P*-value.
[b]Negative Z-scores correspond to predicted decrease in canonical pathway activity; positive z-scores correspond to predicted increase in canonical pathway activity. N/A indicates that IPA did not attempt to assign a Z-score to that pathway and 0 indicates that the pathway was not predicted to be increased nor decreased. Pathways with scores of N/A or 0 therefore do not have predicted activation states listed.
[c]Predicted activation state is calculated by IPA software and indicates whether canonical pathway activity is increased or decreased.
[d]The number of DORs present in IPA's database, corresponding to each canonical pathway.
n = 5 juvenile mCD, n = 5 juvenile mWSD.

Table S7. Upstream regulators predicted from DORs in juvenile HSPCs and BMDMs.

Upstream regulator name[a]	p value	Z-score[b]	Predicted activation state[c]	# DORs in pathway[d]
**HSPCs**				
5-fluorouracil	1.61E-08	-1.45	Up-regulated	24
sirolimus	8.97E-07	0.17	Up-regulated	29
HNF4A	1.27E-06	0.62	Up-regulated	66
trichostatin A	2.05E-06	0.31	Up-regulated	38
E2F1	2.82E-06	1.10	Up-regulated	25
MYC	6.19E-06	3.92	Up-regulated	44
APP	7.81E-06	0.86	Up-regulated	36
**BMDMs**				
KLF6	1.21E-19	4.84	Up-regulated	42
TNF	1.77E-17	4.95	Up-regulated	123
lipopolysaccharide	2.04E-16	4.80	Up-regulated	138
camptothecin	2.85E-16	2.14	Up-regulated	59
IFNG	4.28E-16	4.29	Up-regulated	101
IL4	2.33E-15	0.85	Up-regulated	86
IL1A	2.84E-15	3.62	Up-regulated	35
TGFB1	4.13E-15	-0.59	Down-regulated	117
5-fluorouracil	3.06E-14	0.85	Up-regulated	41
NFE2L2	5.97E-14	0.30	Up-regulated	47
NR3C1	7.05E-14	-2.08	Down-regulated	61
TNFSF11	9.01E-14	3.73	Up-regulated	35
IgG	1.55E-13	0.80	Up-regulated	38
FAS	3.16E-13	0.42	Up-regulated	40
LY294002	3.99E-13	-2.59	Down-regulated	49
IL1B	4.95E-13	4.37	Up-regulated	77
resiquimod	1.18E-12	3.38	Up-regulated	34
NFKBIA	3.23E-12	2.40	Up-regulated	43
IL2	3.93E-12	3.21	Up-regulated	56
trichostatin A	5.09E-12	1.06	Up-regulated	68
TNFRSF1A	5.57E-12	3.02	Up-regulated	25
PD98059	5.69E-12	-2.28	Down-regulated	49
CD40LG	8.15E-12	3.11	Up-regulated	39
beta-estradiol	9.23E-12	0.82	Up-regulated	129

HNF4A	9.63E-12	1.78	Up-regulated	114

[a]Shown in rank order by p value.
[b]Negative Z-scores correspond to predicted decrease in upstream regulator pathway activity; positive Z-scores correspond to predicted increase in upstream regulator pathway activity.
[c]Predicted activation state is calculated by IPA software and indicates whether the upstream regulator pathway activity is decreased or increased.
[d]The number of DORs present in IPA's database, corresponding to each upstream regulator pathway.
n = 5 juvenile mCD, n = 5 juvenile mWSD.

Table S8. Canonical pathways predicted from DORs overlapping in juvenile HSPCs and BMDMs, regulated by mWSD.

Canonical pathway name[a]	-Log(p value)	Z-score[b]	# DORs in pathway[c]
FAT10 Signaling Pathway	3.17	N/A	3
NAD Phosphorylation and Dephosphorylation	3.15	N/A	2
NAD Salvage Pathway II	2.51	N/A	2
TNFR2 Signaling	2.36	N/A	2
NRF2-mediated Oxidative Stress Response	2.22	N/A	4
D-glucuronate Degradation I	2.04	N/A	1
Geranylgeranyldiphosphate Biosynthesis	2.04	N/A	1
Protein Ubiquitination Pathway	2.00	N/A	4
Inhibition of ARE-Mediated mRNA Degradation Pathway	1.88	N/A	3
Tetrapyrrole Biosynthesis II	1.82	N/A	1
Activation of IRF by Cytosolic Pattern Recognition Receptors	1.77	N/A	2
Induction of Apoptosis by HIV1	1.77	N/A	2
CD40 Signaling	1.74	N/A	2
D-myo-inositol (1,4,5,6)-Tetrakisphosphate Biosynthesis	1.74	N/A	3
D-myo-inositol (3,4,5,6)-Tetrakisphosphate Biosynthesis	1.74	N/A	3
IL-17 Signaling	1.71	N/A	3

[a]Shown in rank order by p value.
[b]N/A indicates that IPA did not attempt to assign a Z-score to that pathway. Pathways with scores of N/A therefore do not have predicted activation states listed.
[c]The number of DORs present in IPA's database, corresponding to each canonical pathway.
n = 5 juvenile mCD, n = 5 juvenile mWSD.

Table S9. Upstream regulators predicted from DORs overlapping in juvenile HSPCs and BMDMs, regulated by mWSD.

Upstream regulator name[a]	p value	Z-score[b] (HSPCs)	Z-score[b] (BMDMs)	Predicted activation state[c]	# DORs in pathway[d]
Gnasas1	1.46E-05	-1.98	-1.07	Down-regulated	4
EPHB6	1.73E-05	1.15	1.15	Up-regulated	4
DROSHA	2.11E-05	N/A	N/A		3
5-fluorouracil	2.41E-05	0.18	0.18	Up-regulated	8
camptothecin	3.54E-05	1.00	0.20	Up-regulated	10
L-tryptophan	4.73E-05	N/A	N/A		3
IgG1	4.73E-05	N/A	N/A		3
TGFB1	5.24E-05	0.46	-0.93	Up/Down	18
1,2-dithiol-3-thione	5.69E-05	-0.03	0.17	Down/Up	6
meclofenamic acid ethyl ester	6.12E-05	N/A	N/A		2
SB 290157	6.19E-05	N/A	N/A		3
IL1B	7.18E-05	3.07	1.17	Up-regulated	13

[a]Shown in rank order by p value.
[b]Negative Z-scores correspond to inhibited upstream regulator pathways; positive Z-scores correspond to activated upstream regulator pathways. N/A indicates that IPA software did not attempt to assign an activation state, so regulators with Z-scores of N/A have no predicted activation state.
[c]Predicted activation state is calculated by IPA software and indicates whether the upstream regulator pathway is inhibited or activated. If predicted activation state is different in HSPCs vs. BMDMs, activation states are presented with a slash between them, corresponding to activation state in HSPCs/activation state in BMDMs.
[d]The number of DORs present in IPA’s database, corresponding to each upstream regulator.
n = 5 juvenile mCD, n = 5 juvenile mWSD.

Table S10. Canonical pathways predicted from DEGs regulated by mWSD in fetal HSPCs.

Canonical pathway name[a]	-Log(p value)	Z-score[b]	Predicted activation state[c]	# DEGs in pathway[d]
Production of Nitric Oxide and Reactive Oxygen Species in Macrophages	4.97	1.89	Up-regulated	9
Type I Diabetes Mellitus Signaling	3.78	1.34	Up-regulated	6
iNOS Signaling	3.49	2.00	Up-regulated	4
EIF2 Signaling	2.88	0		7
MSP-RON Signaling in Macrophages Pathway	2.84	-0.45	Down-regulated	5
ERK5 Signaling	2.72	1.00	Up-regulated	4
T Helper Cell Differentiation	2.69	N/A		4
Macropinocytosis Signaling	2.63	N/A		4
Actin Nucleation by ARP-WASP Complex	2.53	N/A		4
IL-12 Signaling and Production in Macrophages	2.53	N/A		5

[a]Shown in rank order by p value.
[b]Negative Z-scores correspond to predicted decrease in canonical pathway activity; positive Z-scores correspond to predicted increase in canonical pathway activity. N/A indicates that IPA did not attempt to assign a Z-score to that pathway and 0 indicates that the pathway was not predicted to be increased nor decreased. Pathways with scores of N/A or 0 therefore do not have predicted activation states listed.
[c]Predicted activation state is calculated by IPA software and indicates whether the canonical pathway activity is increased or decreased.
[d]The number of DEGs present in IPA's database, corresponding to each canonical pathway.
n = 5 fetal mCD, n = 5 fetal mWSD.

Table S11. Upstream regulators predicted from DEGs regulated by mWSD in fetal HSPCs.

**Upstream regulator name**[a]	**p value/-Log(p value)**	**Z-score**[b]	**Predicted activation state**[c]	**# DEGs in pathway**[d]
lipopolysaccharide	1.39E-11	2.65	Up-regulated	45
TGFB1	6.27E-10	2.77	Up-regulated	40
IL2	1.24E-08	2.27	Up-regulated	21
dexamethasone	3.47E-08	1.40	Up-regulated	41
TNF	4.63E-08	2.74	Up-regulated	37
FOS	7.36E-08	0.74	Up-regulated	19
IFNG	1.20E-06	2.52	Up-regulated	29
KLF3	1.33E-06	-1.44	Down-regulated	13
D-glucose	1.41E-06	2.00	Up-regulated	19
IL4	1.77E-06	0.89	Up-regulated	23
IL3	1.85E-06	1.30	Up-regulated	13
TP53	1.92E-06	2.83	Up-regulated	34
IL6	1.93E-06	2.52	Up-regulated	20
CD38	3.40E-06	2.97	Up-regulated	9
interferon beta-1a	4.18E-06	N/A		8
TCL1A	4.57E-06	N/A		7
Immunoglobulin	5.42E-06	-1.69	Down-regulated	18
PD98059	6.95E-06	-2.27	Down-regulated	16

[a]Shown in rank order by p value.
[b]Negative Z-scores correspond to inhibited upstream regulator pathways; positive Z-scores correspond to activated upstream regulator pathways. N/A indicates that IPA software did not attempt to assign an activation state, so regulators with Z-scores of N/A have no predicted activation state.
[c]Predicted activation state is calculated by IPA software and indicates whether the upstream regulator pathway activity is decreased or increased.
[d]The number of DEGs present in IPA’s database, corresponding to each upstream regulator pathway.
n = 5 fetal mCD, n = 5 fetal mWSD.

Table S12. Upstream regulators predicted from the set of DEGs overlapping in both fetal and juvenile HSPCs, regulated by mWSD.

Upstream regulator name[a]	p value	Z-score[b] (fetal)	Z-score[b] (juvenile)	Predicted activation state[c]	# DEGs in pathway[d]
IL9	5.09E-07	-0.28	-0.28	Down-regulated	5
lipopolysaccharide	8.85E-06	0.97	1.57	Up-regulated	19
puromycin aminonucleoside (PAN)	1.12E-05	1.34	1.34	Up-regulated	5
TGFB2	1.51E-05	0.83	1.51	Up-regulated	5
PTP4A1	1.64E-05	0.45	0.45	Up-regulated	5
tretinoin	3.10E-05	1.58	2.90	Up-regulated	15
fenamic acid	3.47E-05	1.13	1.13	Up-regulated	4
TP53	4.12E-05	0.31	0.31	Up-regulated	16
IL1	4.24E-05	0.82	0	Up/Neither	7
indomethacin	5.13E-05	-0.04	-0.04	Down-regulated	6
cerivastatin	5.62E-05	N/A	N/A		4
RACGAP1	6.95E-05	N/A	N/A		2

[a]Shown in rank order by p value.
[b]Negative Z-scores correspond to predicted decrease in canonical pathway activity; positive Z-scores correspond to predicted increase in canonical pathway activity. N/A indicates that IPA did not attempt to assign a Z-score to that pathway and 0 indicates that the pathway was not predicted to be increased nor decreased. Pathways with scores of N/A or 0 therefore do not have predicted activation states listed. Z-scores are separated by fetal and postnatal scores.
[c]Predicted activation state is calculated by IPA software and indicates whether the upstream regulator pathway activity is decreased or increased. If pathway activation state is different in fetal vs. juvenile HSPCs, different states are separated by a slash.
[d]The number of DEGs present in IPA's database, corresponding to each upstream regulator pathway.
n = 5 fetal mCD, n = 5 fetal mWSD; n = 5 juvenile mCD, n = 6 juvenile mWSD.

Table S13. Predicted transcription factor interactions derived from DEGs in fetal and juvenile HSPCs exposed to mWSD.

Interacting transcription factors[a]	Z-score
**Fetal**	
EGR1, EGR2, EGR3 and SP1, SP2, SP3, SP4	5.76
ETS1 and SP1, SP2, SP3, SP4	4.95
CEBPB and STAT6	4.89
FOS, FOSB, FOSL1, FOSL2 and JUN	4.81
MAX, MYC and SP1, SP2, SP3, SP4	4.74
**Juvenile**	
FOS, FOSB, FOSL1, FOSL2 and JUN	5.19
CEBPA, CEBPB, CEBPD, CEBPE, CEBPG and STAT6	4.93
CEBPB and STAT6	4.88
ETS1 and NFKB1, NFKB2, RELA	4.67
ETS1 and EGR1, EGR2, EGR3	4.66
MAX, MYC and AHR, AHRR, ARNT, ARNT2, HIF1A	4.58

[a]Top five interacting transcription factor groups for fetal HSPCs and juvenile HSPCs based on PC-TraFF analysis.
n = 5 fetal mCD; n = 5 fetal mWSD; n = 5 juvenile mCD, n =6 juvenile mWSD.

Table S14. Fetal complete blood counts.

CBC variable	mCD	mWSD	p value
WBC, X 10^9/L	2.7 ± 0.3	3.0 ± 0.7	0.76
Neutrophils, %	10.2 ± 1.6	11.2 ± 2.0	0.71
Lymphocytes, %	80 ± 2.0	81 ± 2.0	0.86
Monocytes, %	8.4 ± 1.2	7.1 ± 0.2	0.35
Eosinophils, %	0.01 ± 0.0	0.02 ± 0.0	0.75
Basophils, %	0.85 ± 0.1	0.60 ± 0.1	0.06
Hematocrit, %	37.3 ± 0.7	36.1 ± 2.2	0.65
Hemoglobin, mmol/L	12.4 ± 0.2	12.0 ± 0.6	0.56
Red blood cells, X 10^{12}/L	3.6 ± 0.0	3.5 ± 0.1	0.61
MCV, fL	102 ± 1.0	101 ± 1.0	0.81
MCH, fmol/cell	34.0 ± 0.5	33.7 ± 0.1	0.61
MCHC, mmol/L	33.3 ± 0.1	33.2 ± 0.2	0.76
Platelets, X 10^9/L	323 ± 23	323 ± 32	0.99

CBC, complete blood count; WBC; white blood cells, MCV; mean corpuscular volume, MCH; mean corpuscular hemoglobin, MCHC; mean corpuscular hemoglobin concentration. % indicates percentage of WBC count. Unpaired Student's t test p values are shown.
n = 8 fetal mCD and n = 5 fetal mWSD.

Table S15. Free fatty acid concentrations in fetal bone marrow and liver and juvenile bone marrow.

FFA species	mCD	mWSD	p value
**Fetal bone marrow**			
Palmitic acid	5771 ± 923	5418 ± 350	0.73
Stearic acid	4180 ± 972	3128 ± 113	0.33
Oleic acid	3651 ± 525	6237 ± 307	**<0.005**
Linoleic acid	1598 ± 315	1549 ± 271	0.9
Linolenic acid	125 ± 18	75 ± 6	**<0.05**
**Fetal liver**			
Palmitic acid	30076 ± 5863	33221 ± 9541	0.78
Palmitoleic acid	2325 ± 535	3827 ± 980	0.23
Stearic acid	23384 ± 4585	25778 ± 10043	0.83
Oleic acid	22757 ± 4614	45119 ± 7722	**0.05**
Linoleic acid	19024 ± 4407	23135 ± 8287	0.68
Linolenic acid	450 ± 93	192 ± 36	**<0.05**
Arachidonic acid	5305 ± 3319	13483 ± 10485	0.5
**Juvenile bone marrow**			
Palmitic acid	7730 ± 976	11655 ± 2436	0.17
Stearic acid	3229 ± 263	3783 ± 567	0.39
Oleic acid	9651 ± 1595	16564 ± 2803	**0.05**
Linoleic acid	14494 ± 3108	29566 ± 5327	**<0.05**
Linolenic acid	1319 ± 245	2324 ± 515	0.11

Data are presented as mean ± SEM. Units are μg/mL. Unpaired Student's t test p values are shown (significant p value in bold). n = 6-7 fetal mCD, n = 4-5 fetal mWSD; n = 8 juvenile mCD, n = 7 juvenile mWSD.
FFA, free fatty acid.

Table S16. Eicosanoid concentrations in fetal bone marrow and liver and juvenile bone marrow.

Eicosanoid species	mCD	mWSD	p value
**Fetal bone marrow**			
TXB2	4.9 ± 0.7	2.8 ± 0.5	**<0.05**
PGE2	2.5 ± 0.3	1.3 ± 0.2	**<0.05**
6-Keto-PGF1a	4.6 ± 0.7	4.2 ± 0.6	0.75
PGF2a	2.0 ± 0.6	0.7 ± 0.3	0.13
5-HETE	0.92 ± 0.08	0.71 ± 0.36	0.6
12-HETE	340 ± 45	329 ± 49	0.87
15-HETE	1.7 ± 0.2	1.0 ± 0.1	0.063
AA	524 ± 77	276 ± 29	**<0.05**
DHA	857 ± 109	374 ± 84	**<0.05**
**Fetal liver**			
TXB2	0.11 ± 0.02	0.04 ± 0.01	0.06
PGE2	0.81 ± 0.25	0.30 ± 0.11	0.1
6-Keto-PGF1a	0.13 ± 0.06	0.04 ± 0.03	0.22
PGF2a	0.20 ± 0.06	0.04 ± 0.03	0.059
5-HETE	1.6 ± 0.7	2.1 ± 0.7	0.65
12-HETE	9.4 ± 5.9	6.3 ± 1.6	0.63
15-HETE	2.6 ± 0.7	2.3 ± 0.6	0.79
AA	3455 ± 854	2788 ± 748	0.57
DHA	6350 ± 1369	5472 ± 1235	0.64
EPA	108 ± 25	24 ± 9	**<0.05**
**Juvenile bone marrow**			
TXB2	5.5 ± 0.4	4.1 ± 0.4	**<0.05**
PGE2	2.3 ± 0.2	3.2 ± 0.9	0.42
6-Keto-PGF1a	1.0 ± 0.1	1.3 ± 0.4	0.52
PGF2a	2.1 ± 0.3	1.9 ± 0.4	0.7
5-HETE	2.2 ± 0.5	3.8 ± 1.3	0.28
12-HETE	168 ± 20	183.8 ± 41	0.74
15-HETE	2.0 ± 0.3	2.4 ± 0.3	0.47
AA	581 ± 85	670 ± 101	0.51
DHA	2035 ± 377	2423 ± 326	0.44
EPA	0.4 ± 0.1	0.6 ± 0.1	0.59

Data are presented as mean ± SEM. Units are μg/mL. Unpaired Student's t test p values are shown (significant p value in bold). n = 6-7 fetal mCD, n = 4 fetal mWSD; n = 9 juvenile mCD, n = 7 juvenile mWSD.
